# Which demographic processes control competitive equilibria? Bayesian calibration of a size‐structured forest population model

**DOI:** 10.1002/ece3.10232

**Published:** 2023-07-04

**Authors:** Lukas Heiland, Georges Kunstler, Vladimír Šebeň, Lisa Hülsmann

**Affiliations:** ^1^ Bayreuth Center of Ecology and Environmental Research (BayCEER), Ecosystem Analysis and Simulation (EASI) Lab University of Bayreuth Bayreuth Germany; ^2^ Theoretical Ecology University of Regensburg Regensburg Germany; ^3^ Université Grenoble Alpes, Inrae, LESSEM Grenoble France; ^4^ Národné lesnícke centrum Zvolen Slovakia

**Keywords:** dynamic model, *Fagus sylvatica*, monodominance, national forest inventory, natural regeneration, sapling competition, species interactions

## Abstract

In forest communities, light competition is a key process for community assembly. Species' differences in seedling and sapling tolerance to shade cast by overstory trees is thought to determine species composition at late‐successional stages. Most forests are distant from these late‐successional equilibria, impeding a formal evaluation of their potential species composition. To extrapolate competitive equilibria from short‐term data, we therefore introduce the JAB model, a parsimonious dynamic model with interacting size‐structured populations, which focuses on sapling demography including the tolerance to overstory competition. We apply the JAB model to a two‐“species” system from temperate European forests, that is, the shade‐tolerant species *Fagus sylvatica* L. and the group of all *other* competing species. Using Bayesian calibration with prior information from external Slovakian national forest inventory (NFI) data, we fit the JAB model to short time series from the German NFI. We use the posterior estimates of demographic rates to extrapolate that *F. sylvatica* will be the predominant species in 94% of the competitive equilibria, despite only predominating in 24% of the initial states. We further simulate counterfactual equilibria with parameters switched between species to assess the role of different demographic processes for competitive equilibria. These simulations confirm the hypothesis that the higher shade tolerance of *F. sylvatica* saplings is key for its long‐term predominance. Our results highlight the importance of demographic differences in early life stages for tree species assembly in forest communities.

## Graphical abstract

Here, we present a forest population model with species interactions and explicit representation of the sapling stage, to extrapolate long‐term competitive equilibria among tree species from short‐term time series. Fitting our model to national forest inventory (NFI) data from Germany and Slovakia, we show for the first time with simulations from a demographic model, that because of its shade‐tolerant saplings, common beech (*Fagus sylvatica* L.) will be the naturally predominating tree species in Central European forests.

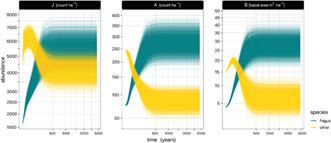

## 1 INTRODUCTION

Among the species interactions that shape community composition, competition is one of the most important in forest ecosystems (Goldberg & Barton, 1992; Tilman, 1982). While in recent years, the role of facilitation in species assembly has been increasingly acknowledged (Bruno et al., 2003; Pretzsch et al., 2013; Simha et al., 2022), trees in forests are subject to exceptional levels of competition for light. Tree species are in an evolutionary arms race with other plants and acquired slowly growing woody structures as a means of increasing their access to light with increasing height (Clements et al., 1929; Givnish, 1988; Keddy, 2001). Not only does the upper hand in height lead to an advantage for assimilating carbon that is size symmetric, but also harvesting light that comes from above means excluding trees below, which is a size‐asymmetric competitive advantage for larger trees (Schwinning, 1998). Hence, the differential ability of tree recruits to tolerate the shading effect of the competing overstory is a key determinant of tree species assembly in forests (Emborg, 1998), in addition to competitiveness for belowground resources such as water and nutrients (Coomes & Grubb, 2000; Putz & Canham, 1992).

Evidence from around the world suggests that high shade tolerance of saplings is a mechanism for individual tree species to attain predominance in forests at the competitive equilibrium, that is, the equilibrium of species abundances as the result of interacting demographic processes in the absence of major disturbances (Pickett, 1980). Instances, where species with shade‐tolerant saplings tend to be predominant in late‐successional forests are common across climate zones and clades, for example, *Tsuga canadensis* (L.) carrière in temperate forests (eastern North America; Rogers, 1978; see also Canham, 1989), *Sloanea woollsii* F. muell. in subtropical rainforests (eastern Autralia; Baur, 1957; Floyd, 1990), and *Gilbertiodendron dewevrei* de wild. in tropical lowland forests (Ituri Forest of Zaïre; Hart, 1995). Shade‐tolerant tree species, whose recruits are viable under a closed canopy over long periods, are generally contrasted with “pioneer” species that are only able to regenerate after a disturbance has opened the canopy (Whitmore, 1989). These different recruiting strategies highlight the crucial importance of the sapling stage for competitive exclusion, which can finally lead to predominance at the competitive equilibrium.

In temperate European forests, *Fagus sylvatica* L. (European beech, hereinafter abbreviated “*Fagus*”) is traditionally thought to be the shade‐tolerant tree species that dominates at competitive equilibrium (Ellenberg, 1963; Watt, 1923). *Fagus* saplings can survive particularly well under shading (Petrovska, Brang, et al., 2021), so that *Fagus* is thought to naturally predominate, at least in environments at submontane elevations without higher abiotic stress or major disturbances (Ellenberg, 1963). That *Fagus* would potentially predominate in Central European forests, has however not been tested with data at a large scale. This kind of test is impeded by the fact that in reality the Central European landscape has been altered by humans, ever since *Fagus*' ongoing post‐glacial immigration (Lang, 1994; Magri, 2008), so that *Fagus* is not de facto the predominant tree species: Knapp (2008) estimates that currently only 4.5% of Germany is covered with beech forests, in contrast to their projected natural cover of about 66%.

Given that only data from disturbed and managed forests are available, models can be used to project which species will predominate at the competitive equilibrium. Not all models, however, are suited to the task: Along the spectrum between correlative (statistical) and process‐based models (Dormann et al., 2012), purely correlative models have the advantage that they can be easily fitted to data. Correlative models, however, have the disadvantage that they cannot be used for extrapolating states outside the domain of the data, and furthermore, they can only rarely be used for inference on the underlying processes (Dormann et al., 2012; Korzukhin et al., 1996). On the other extreme, process‐based dynamic vegetation models can extrapolate potential community states outside the data domain by assuming that the same processes act universally, but are often hard to fit to data as they require a multitude of parameters to be calibrated (Hartig et al., 2012; Korzukhin et al., 1996). Several modeling attempts have been undertaken to extrapolate the equilibrium forest vegetation with relatively complex process models that concentrate on larger trees, but neither explicitly consider the role of the sapling stage nor quantify the role of sapling demography (e.g., Badeck et al., 2001; Bugmann & Solomon, 2000; Prentice et al., 1993). However, the critical role of recruitment processes in determining forest composition has recently led to calls for explicit modeling of these processes (Hanbury‐Brown et al., 2022; König et al., 2022; Kunstler et al., 2009; Price et al., 2001).

Here, to extrapolate long‐term competitive equilibria from short‐term forest dynamics, we propose the JAB model, a simple dynamic population model that includes species interactions and a sapling stage. The JAB model explicitly represents the species‐specific response of saplings to competition from the overstory with a two‐layer size structure (e.g., as in Cordonnier et al., 2019; Lundqvist, 1995). Besides the competition response to the overstory, the sapling dynamics in the model include key demographic processes, like competition effects among saplings, growth, and seedling recruitment. In contrast, the competition between trees of the overstory is only represented by a simple density‐dependent population model. Thus, being based on demographic processes but decisively parsimonious, the JAB model combines the advantages of the correlative and process‐based modeling: It is relatively easy to fit to data while still being suited for predicting competitive equilibria (see also Clark et al., 2020) and inferring the role of individual demographic processes (see also Briscoe et al., 2019).

We apply the JAB model by fitting it to data from the German national forest inventory (NFI), using Bayesian calibration with prior parameter distributions based on external information from the Slovakian NFI data. For the question at hand, “will *Fagus sylvatica* be predominant at the competitive equilibrium?,” we group all *other* tree species into one population and let them compete with *Fagus* to project the population states at the competitive equilibria. To test the hypothesis that the higher tolerance of *Fagus* saplings to competition from the overstory is key in its predominance, we simulate and compare the predominance in counterfactual competitive equilibria where demographic parameters have been switched between *Fagus* and *others*.

## 2 METHODS

Here, we present the JAB model (Section 2.1) and the NFI data that were used for its calibration (Section 2.2 and Appendix A.1). Using a Bayesian calibration approach, we facilitated the fit of the model by constraining the seedling recruitment rates with prior parameter distributions, which were inferred by regression with auxiliary data from the Slovakian NFI (Section 2.3 and Appendix A.4). With the inferred priors, we used an observation model to fit the JAB model to count data (Section 2.4), linking the area‐standardized basal area states in the model to angle count data with basal area‐related offsets (Section 2.2.2 and Appendix A.2). Finally, we simulated data from the fitted JAB model for inference about competitive equilibria and their drivers (Section 2.5).

All data analyses were performed with R (version 4.0.5; R Core Team, 2021). All models have been written in the language stan (version 2.30.1; Stan Development Team, 2022) and were fitted with the package cmdstanr (version 0.5.3; Gabry & Češnovar, 2021) and the default Hamiltonian Monte Carlo (HMC) algorithm (Section 2.6).

### 2.1 JAB model

**FIGURE 1:**
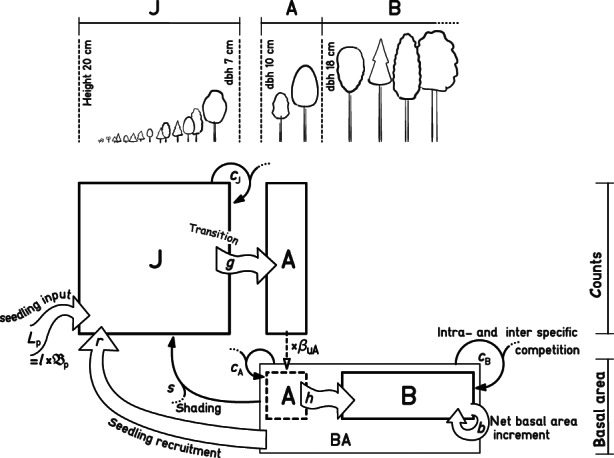
The JAB model represents three different size stages of a tree population (J, A, B) and demographic processes, including the competitive interactions between size stages and multiple species. There are processes that contribute to population growth: seedling input into the population from temporal or spatial dispersal *L*_p_ (dependent on regional basal area $B_{p}$), within‐population seedling recruitment *r*, transitions from a smaller to a larger stage (*g*, *h*), and the net basal area growth *b*. The processes that limit population growth comprise the competition effect from the total sum of the sapling stage J on species‐specific J (*c*_J_), the competition from the sum of A and B, that is, the total basal area of all species BA, on A and B (*c*_A_, *c*_B_), as well as the asymmetric competition from BA on J (“shading” effect *s*). The choice of thresholds between size stages is informed by the size classes in the data. In the German NFI, small trees between height 20 cm and dbh 7 cm were counted, while trees with dbh >10 cm were additionally measured in terms of basal area, so that intermediary size stage A acts as a mediator between count data for J and the basal area data for B. The factor $\beta_{\mathrm{uA}}$, that is, the upper basal area of a tree in A, converts A from counts to basal area.

The JAB model is a dynamic model with competition effects that includes an explicit juvenile stage (Figure 1; for code see Appendix B). The JAB model describes populations of species that are logistically limited by the same resource (Gause, 1932) but differs from a classical competitive Lotka–Volterra model in four main aspects: (1) size‐structured populations, (2) basal area growth, (3) reduced complexity of competition effects, and (4) influx from outside the populations.

In the JAB model, populations are structured into three size stages that interact: the juvenile stage J, representing the understory, and the stages A and B, jointly representing the overstory (Figure 1). Partitioning tree populations into understory and overstory, we can express asymmetric competition between the two fundamentally different forest layers (Schwinning, 1998): the understory is affected by the shading of the overstory, while the overstory is directly exposed to light and unaffected by the understory (Angelini et al., 2015; Cordonnier et al., 2019; De Lombaerde et al., 2019; Valladares & Niinemets, 2008). The overstory (BA) is divided into A and B to enable conversion between measures of tree abundance in the sapling stage J, which is quantified as a count density, and the final stage B, which is represented in terms of basal area. The count density in J reflects the common measure of sapling inventories in NFI (see Appendix A.1.1), whereas the basal area in the overstory stage B is a common measure for timber growth and competition (Biging & Dobbertin, 1992). The size stage A functions as an intermediary between J (only counts) and B (only basal area) by having counts that are converted to basal area with a conversion factor. The JAB model represents growth as transition rates in absence of competition: from the understory stage J to the intermediary stage A (parameter *g*) and from A to B (parameter *h*). In addition to transitions from A, the final stage B has intrinsic basal area growth (parameter *b*; Table 1).

**TABLE 1:** Parameters of the JAB model and their explanation.

Stage	Symbol	Shorthand	Explanation	Effect from …	On	(Direction)
J	*l or L_p_*	Input	Seedling input from large‐scale and long‐term basal area average to specific J	B	J	+
	*r*	Recruitment	Seedling recruitment from the local basal area to specific J	BA	J	+
	*c*_J_	Juvenile competition	Competition effect from total count of juveniles on specific J	sum J	J	−
	*s*	Shading	Competition effect from total basal area on specific J	sum BA	J	−
J/A	*g*	Transition to A	Transition due to survival and growth from specific J to specific A	J/A	A/J	+/−
A	*c*_A_	Competition on A	Competition effect from total basal area on specific A	sum BA	A	−
A/B	*h*	Transition to B	Survival and growth from specific A to specific B	A/B	B/A	+/−
B	*b*	Net basal area growth	Net basal area increment of specific B dependent on specific B (also includes mortality)	B	B	+
	*c*_B_	Competition on B	Competition effect from all basal area on specific B (includes both mortality and growth reduction)	sum BA	B	−

*Note*: The parameters generally link two model states, so that there is an effect acting from some model state on another that can be positive or negative (indicated in column “Direction”).

To reduce the complexity of competition compared to a full Lotka–Volterra model, we represented only the differences in species' response to competition, assuming a similar competition effect among species (simplifying from a matrix of *n* ^2^ parameters to parameter vectors of *n* competing species). More specifically, species are affected by the competition from the sum of the basal area of all species within their respective layers, that is, they have a different competitive response to the sum of all inter‐ and intraspecific competition (vectors of species‐specific parameters *c*_J_, *c*_A_, and *c*_B_). In applying this competition structure, we assume that the difference in competitive response between species is much more important than their difference in competitive effect (Goldberg, 1996; Goldberg & Landa, 1991; Tilman, 1982). The asymmetric competition from the overstory BA on J is represented by the “shading” parameter *s*.

In the JAB model, there are two different sources of seedling recruitment: (1) local recruitment that is proportional to the local conspecific basal area (parameter *r*) and (2) external seedling input. The external seedling input *L*_p_ represents all long‐term persistence of diaspores and long‐distance dispersal into a subpopulation that is not explained by the local conspecific basal area of a plot *p*. It is proportional to a measure of long‐term and large‐scale distribution of the species $B_{p}$ with the coefficient and parameter *l* (detailed in Appendix A.3).

**FIGURE 2:**
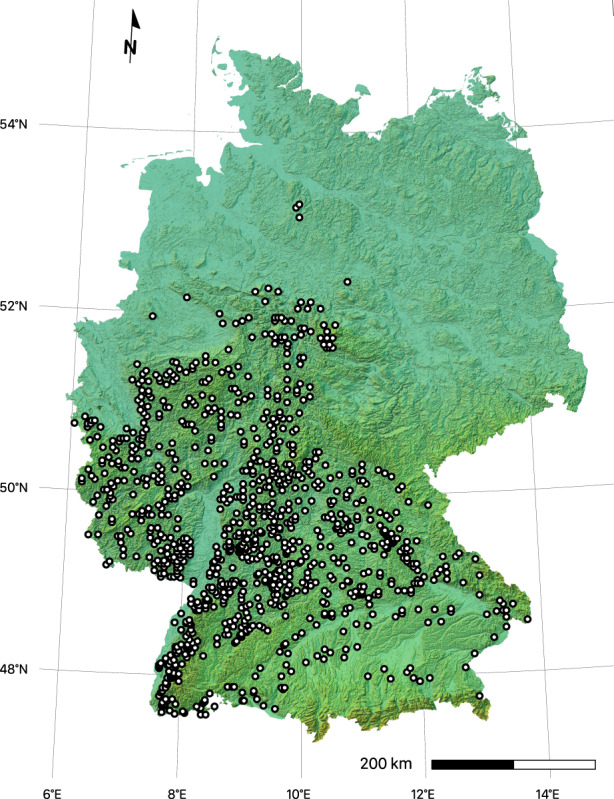
Locations of the 1000 randomly selected NFI clusters in Germany. Each cluster consists of one to four sampling plots, depending on whether the location was forested. One random plot per cluster was selected if its elevation was at 100–600 m, if there was any observation of both *Fagus* and *others*, if the observation period included all of the three surveys (1987, 2002, and 2012; which excludes former East Germany), and if there were no records of management within the period. Each selected plot (cluster) is represented by one subpopulation in the JAB model. The map projection is Lambert Conformal Conic, the color shading indicates elevation and relief.

Based on these principles, we implemented the JAB model as a discrete‐time iteration rule in stan, with hyperbolic density dependence (see Ellner, 1984; Levine & Rees, 2004; Watkinson, 1980), similar to a Lotka–Volterra‐type model formulation in Din (2013). The iteration rule comprises a set of four equations (Equations (1), (2), (3), (4)) that relate states at year t+1 to states at year *t*. Here, in accordance with the software implementation, we provide a vectorized formulation of the model, where all variables, including the parameters, and the stages J [ha^−1^], A [m^2^ ha^−1^], B [m^2^ ha^−1^], and BA [m^2^ ha^−1^] are vectors with length *n* (number of species). These vectors are operated on with element‐wise multiplication ⊙ and division ⊘; the operator sum reduces the stages to a scalar, representing the total abundance of a stage across species (Figure 2).

(1) $$J_{t+1}=L_{p}+r⊙{\mathrm{BA}}_{t}+\left(J_{t}-g⊙J_{t}\right)⊘\left[1+c_{J}\;\mathrm{sum}\left(J_{t}\right)+s\;\mathrm{sum}\left({\mathrm{BA}}_{t}\right)\right]$$

(2) $$\begin{array}{cc} A_{t+1} & =g⊙J_{t}⊘\left[1+c_{J}\;\mathrm{sum}\left(J_{t}\right)+s\;\mathrm{sum}\left({\mathrm{BA}}_{t}\right)\right]\\ & +\left(A_{t}-h⊙A_{t}\right)⊘\left[1+c_{A}\;\mathrm{sum}\left({\mathrm{BA}}_{t}\right)\right] \end{array}$$

(3) $$B_{t+1}=h⊙A_{t}\cdot \beta_{\mathrm{uA}}⊘\left[1+c_{A}\;\mathrm{sum}\left({\mathrm{BA}}_{t}\right)\right]+\left(1+b\right)⊙B_{t}⊘\left[1+c_{B}\;\mathrm{sum}\left({\mathrm{BA}}_{t}\right)\right]$$

(4) $${\mathrm{BA}}_{t+1}=A_{t+1}⊙\beta_{\mathrm{mA}}+B_{t+1}$$

All parameters ($r,c_{J},s,g,c_{A},h,b,c_{B}$, and $L_{p}=lB_{p}$) are generally assumed to be positive, so that all model states are strictly positive at any time (in fitting the model, this will be ensured by exponentiating the parameters sampled on a log‐scale; Section 2.4.1). The four equations, representing size stages, are coupled through states of other stages, so that changes in one state propagate in discrete time steps, for example, from BA to J to A to B. The fractions of trees that survive and grow in the absence of competition from J to A and from A to B are expressed by the transition rates *g* and *h* ($\in \left(0,1\right)$), respectively. The counts in A are transformed to basal area at two different occasions: (1) In the transition to B, the counts are converted by factoring in the basal area of one tree at the threshold between A and B, a species‐independent scalar factor $\beta_{\mathrm{uA}}$, which is dependent on the threshold diameter at breast height (dbh; Equation 3); (2) the combined basal area BA is calculated by multiplying the counts within A with a vector of the corresponding mean basal areas $\beta_{\mathrm{mA}}$, which are species‐specific constants from the data (Equation 4; *Fagus*: 0.01586 m^2^; *others* 0.01611 m^2^).

All stages (J, A, and B) are logistically limited by interspecific tree density: The sapling stage J is limited by the competitive effect from the total basal area across species BA (*s*) and from the total counts within the same stage (*c*_J_), while the stages A and B are only limited by the competitive effect of BA (*c*_A_ and *c*_B_). This limitation is implemented by dividing the states with a denominator that is slightly greater than 1: $\left[1+c_{J}\;\mathrm{sum}\left(J_{t}\right)+s\;\mathrm{sum}\left({\mathrm{BA}}_{t}\right)\right]$ and $\left[1+c_{A,B}\;\mathrm{sum}\left({\mathrm{BA}}_{t}\right)\right]$, for understory and overstory, respectively. Not only the stage abundances are limited, but also the growth processes are density dependent. The growth rates under competition are expressed by the density‐dependent terms $g⊘\left[1+c_{J}\;\mathrm{sum}\left(J_{t}\right)+s\;\mathrm{sum}\left({\mathrm{BA}}_{t}\right)\right]$, $h⊘\left[1+c_{A}\;\mathrm{sum}\left({\mathrm{BA}}_{t}\right)\right]$, and $b⊘\left[1+c_{B}\;\mathrm{sum}\left({\mathrm{BA}}_{t}\right)\right]$, respectively (Table 2).

Overall, this leads to a system of populations that are in an arms race from a disturbed state towards a competitive equilibrium: Depending on their seedling recruitment (*L*_p_ and *r*), through transition (*g*, and *h*) and net basal area increment (*b*) populations can intrinsically only grow or stagnate. This assumes that density‐independent mortality is negligible in J and A, and that density‐independent mortality in B is included in *b* and does not exceed basal area growth in the long term. Thus, the model is built on the assumption that populations of *Fagus* and *others* are viable in the given environmental range. Although, populations can never go extinct, they can decline through interspecific competition (*c*_J_, *s*, *c*_A_, and *c*_B_). These properties enable the JAB model to extrapolate species composition under the assumption that species composition is mainly determined by a competitive equilibrium (Ellenberg, 1963).

### 2.2 NFI data

To fit the JAB model to observed tree populations of *Fagus* and *others*, we used data from two different NFIs: the Slovakian NFI with one survey (2015–2016) and the German NFI with three repeated surveys (main years 1987, 2002, 2012; Table S1).

Data from the Slovakian NFI, which include observations of seedlings with height 10–20 cm, were used to infer prior probability distributions for the seedling recruitment rate *r* (for details on the Slovakian NFI data see Appendix A.1.2).

Subsequently, the short‐term time series data from the German NFI were used to fit the JAB model to repeated observations of aggregated populations (for details on the German NFI data see Appendix A.1.1 for locations of selected plots see Figure 2).

#### 2.2.1 Tree size classes J, A, and B

To calibrate the JAB model with data from the German and the Slovakian NFI, trees were grouped into size classes corresponding to the stages of the JAB model. The stage J included all trees between height 20 cm and dbh 7 cm in the German NFI (Table S2). For stages A and B represented in basal area in the JAB model, we included all trees with a dbh above 10 cm, which is the lower size threshold for the angle count sampling in the German NFI. The threshold between A and B was set to dbh 18 cm. Because the lower dbh threshold of angle counts, and correspondingly the upper threshold for sapling counts, was changed from 10 cm in 1987 to 7 cm in 2002 in the German NFI, counts for saplings of this size were not available for all NFI years creating a size gap between J and A. Despite this gap, in the JAB model fit, we treat the count data in size class J as a proxy for all trees between height 20 cm and dbh 10 cm. For inferring the seedling recruitment rate from the Slovakian NFI, the basal area was summed up over the same size classes A and B (BA) and was used as a predictor for the count density of a size class right below J, seedlings of height 10–20 cm.

For analyzing the potential predominance of *Fagus sylvatica* with the JAB model, trees were split up into *F. sylvatica* and all *others*, a binary classification for which we will use “species” as a shorthand. Within species and size class, stage abundances were calculated per plot by summing up the counts in J and A, and adding up the basal area in A and B.

#### 2.2.2 Accounting for varying sampling areas

To model tree abundances recorded with varying sampling areas using a count process in the likelihood of the JAB model (Section 2.4.2) as well as in the models for prior inference (Section 2.3), we used different offsets. In general, the offsets *o* convert the model states $\hat{x}$, which are abundances standardized per hectare, to the scale of observed counts *x*:

(5) $$x∼o\;\hat{x}$$

Different offsets were used depending on the type of the abundances in the model and the corresponding data: for the offsets that scaled the basal area states in the model to count data from the angle count method see Appendix A.2.1; for the offsets that scaled counts in the model to counts on different fixed areas see Appendix A.2.2. Because all NFI sampling protocols made observation areas dependent on size or abundance, the offsets for zero observations had to be derived separately (see Appendix A.2.3).

These offsets account for the varying sampling intensity that also affects the variance of observations. Furthermore, using a count process with offsets is (1) preferable over modeling a continuous response for the basal area because it reflects the actual observation process with discrete numbers of trees (even when being multiplied with a tree‐specific basal area), and (2) preferable over upscaling the small sampling areas to a common area because this would break distributional assumptions by deflating small counts in the data, for example, when a plot size is one fourth of the common standard area 1 hectare, the smallest measured count per hectare would be 4.

### 2.3 Prior parameter distributions

Prior to fitting the JAB model to aggregated population data from the German NFI, we estimated priors from several sources. First, we used external information from Slovakian NFI data to directly estimate species‐specific uncertainty distributions for seedling recruitment *r* (Table S5). The regression method for inferring priors on *r* from the density of small seedlings with height 10–20 cm in the Slovakian NFI is detailed in Appendix A.4. The uncertainty of the estimated species‐specific parameter distributions was expanded with a factor (see Appendix A.4) and then used as priors to constrain the model. Second, in addition to the estimated species‐specific priors, we specified vague and species‐unspecific priors for all other parameters of the JAB model (Section 2.3.2). Further, we specified several regularizing priors for technical parameters, detailed in Section 2.4. For parametric specifications of prior distributions for the model parameters (Table 2).

#### 2.3.1 Propagating the estimated priors

We propagated the fitted posterior parameter distributions for the species‐specific recruitment rate *r* (see Appendix A.4) as priors in the JAB model fit. We first visually checked the posteriors for normality and then fitted a closed‐form maximum likelihood estimate of the normal distribution (fitdistr() in the R package MASS version 7.3; Ripley, 2022) to the posterior HMC samples of species‐specific log *r*. To express the additional uncertainty due to historical and geographical differences in Slovakia (see Appendix A.1.2), the fitted standard deviations of log *r* were multiplied with a factor 4. Finally, the multiplied standard deviations, together with the means of the distributional fits, were passed on to species‐specific normal priors of the parameter log *r*.

#### 2.3.2 Other vague priors for model parameters

**FIGURE 3:**
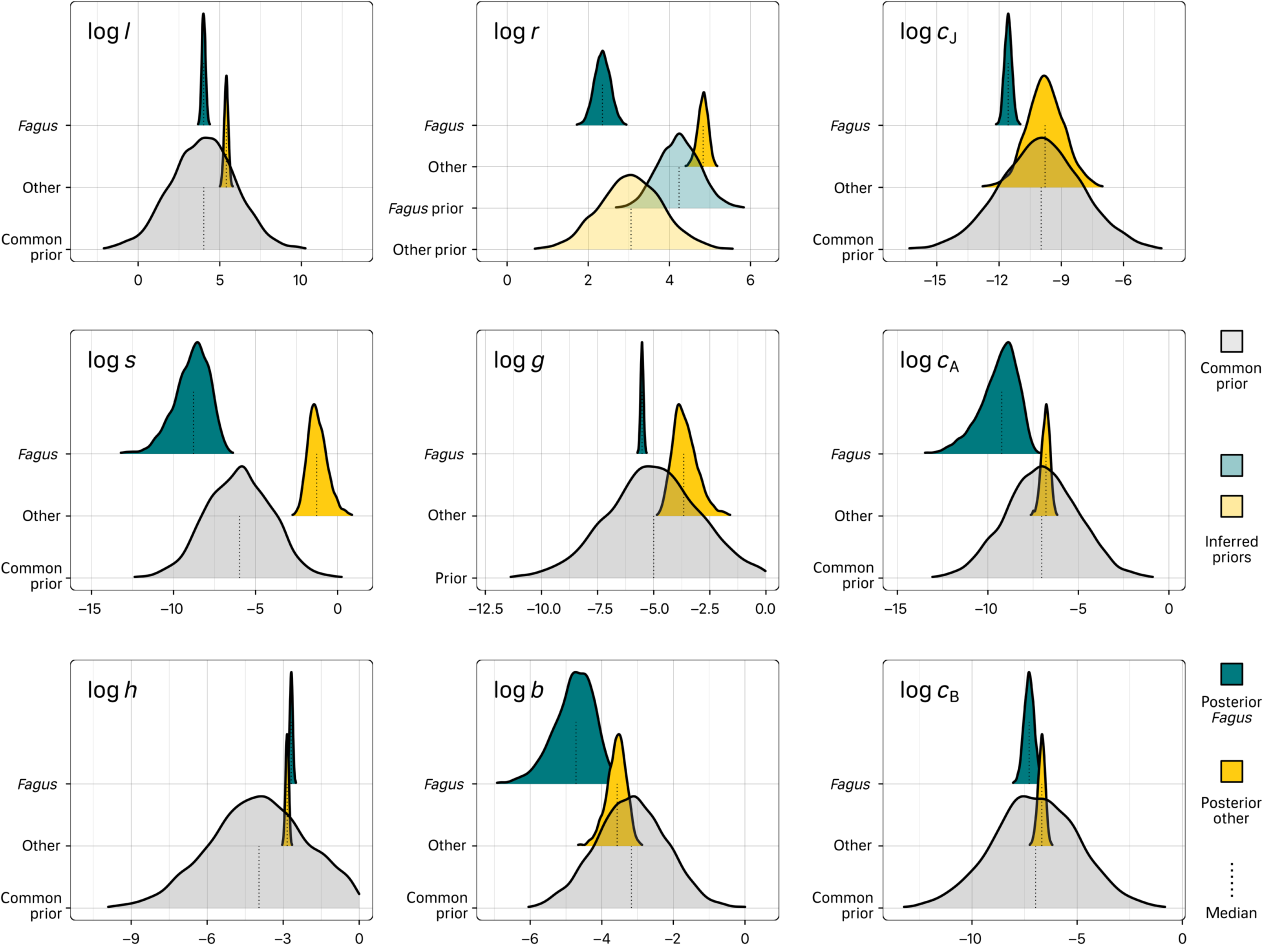
Marginal uncertainties of JAB model parameters including prior (transparent) and posterior distributions (solid) for *Fagus* and *others*. Prior to the JAB model fit, species‐specific distributions for the seedling recruitment parameter logr were inferred with regression models from Slovakian NFI data and propagated as species‐specific normal priors into the fit of the JAB model. The priors for all other parameters (logl, $\log c_{J}$, logs, logg, $\log c_{A}$, logh, logb, and $\log c_{B}$) were specified as common normal priors for both species (Section 2.3.2).

In addition to the species‐specific priors on seedling recruitment that were directly inferred from auxiliary data (log *r*), vague and species‐unspecific normal priors were provided for all other log‐transformed parameters of the JAB model (logl, $\log c_{J}$ logs, logg, $\log c_{A}$, logh, logb, and $\log c_{B}$; Table 2). These priors were specified (1) to improve the convergence of the model fit by reducing the parameter space, (2) in accordance with prior predictive checks of the resulting stage abundances, and (3) to express some prior beliefs: The limiting parameters $\log c_{J}$, $\log c_{A}$, and $\log c_{B}$ express the expectation that the competition response of J to J *c*_J_ is relatively small and that it increases for larger trees parameters *c*_A_ and *c*_B_. The belief that “shading” logs has a stronger effect on J than *c*_J_ was expressed with a vague prior (μ=−6, σ=2). Priors for transition rates express the belief that the fraction of ingrowth relating to saplings J (logg: μ=−5, and sigma=2) is smaller than ingrowth relating to the narrow size class A (logh: μ=−4, and sigma=2). The prior for logb was based on the yearly net basal area increment estimated for surviving trees from the German NFI by (Ruiz‐Benito et al., 2014) of approximately 4% (μ=log0.04≃−3.2) and thus specified with higher confidence (σ=1). The seedling input rate *l* was assumed to be distributed around $\exp\left(4\right)$, which is roughly in line with the seedling input that was not explained by conspecific basal area in the regression on Slovakian data (see Appendix A.4 and Table S5), where the intercept $\exp\left(6\right)≲k≲\exp\left(7\right)$ divided by the average basal area of the respective species (Table 3 is about $\exp\left(4\right)$). Importantly, these priors were equally applied to both species, so that there is only a conservative bias regarding inference about differences between species. For both the vague and species unspecific, as well as the inferred species‐specific priors (see Figure 3 and Table 2).

### 2.4 Bayesian calibration of the JAB model

#### 2.4.1 Model structure

Each sample plot from the German NFI is represented as a subpopulation that changes over time in the JAB model. Each subpopulation has unknown random initial states of the three stages J, A, and B, which are fitted to the first observed state of each plot, and then simulated forward with the JAB model. The JAB model parameters (Table 1) are sampled globally, that is, it is assumed that the simulations of all subpopulations are generated from parameters with one global uncertainty distribution, respectively. These parameter uncertainty distributions are sampled on the log‐scale, informed by the corresponding priors (Section 2.3), and transformed with an exp() statement to be strictly positive, before they are plugged into the JAB model (Equations (1), (2), (3), (4)). The seedling input parameter *l* has subpopulation specific values, $L_{p}=\exp\left(\log l\right)B_{p}$ iterated over the subpopulations or NFI plots *p*. This way, the regional basal area $B_{p}$ informs the subpopulation‐specific seedling input *L*_p_, plugged into Equation 1.

To improve convergence of the model, gamma‐distributed priors were specified for the unknown initial states per subpopulation. These priors express our belief that the initial states are close to the observed value in the data and avoid model fits where the initial states would significantly deviate from the data to compensate for the model dynamics due to extreme parameter combinations. The gamma distributions were parameterized with mode ν and standard deviation ζ,

(6) $$\beta =\frac{\nu +\sqrt{\nu^{2}+4\zeta^{2}}}{2\zeta^{2}}$$

(7) α=1+νβ.

By specifying the mode, we set the highest probability to the observed value, and different standard deviations σ per stage dependent on the observed count κ so that $\sigma =\left[J:\;100+20\kappa ,\;A:\;10+2\kappa ,\;B:1+0.2\kappa \right]$. Cases with zero observations were parameterized with expected value E=α/β and shape α. By setting α=1 and *E* to [J: 0.1, A: 0.02, B: 0.01] times the minimum observed value within that stage, we assumed a shape with the maximum probability always near 0 and different degrees of uncertainty for unobserved trees.

#### 2.4.2 Likelihood function

The JAB model was fitted to two kinds of data, both from count processes: (1) Count data in the size classes J and A originate from a count process on varying areas per tree size, so that the area‐standardized model state (counts per hectare) is related to the observations with an offset *o*, which is the average area of one sampling plot (see Appendix A.2). (2) Basal area data in size class B also originate from a count process of individual trees with varying areas, but in addition, each tree originally had an individual basal area record. Hence, to relate the model state of B (basal area per hectare) to observed counts in size class B, we used a basal area‐related offset that includes both the average sampling area and a factor $c_{p}/{\mathrm{ba}}_{p}$, which expresses how many counts *c*_p_ are added per unit of basal area ${\mathrm{ba}}_{p}$ on average per plot (see Appendix A.2.1). The offsets *o* transform the area‐standardized and strictly positive model state (JAB) to the counts *C* in the data, so that for each plot *p* within a subpopulation *c*, year *t* (or survey *r*), species *j*, and stage *s*, the likelihood function is expressed by the statement:

(8) $$C_{\text{ptjs}}∼\text{NegativeBinomial}\left({\mathrm{JAB}}_{\mathrm{tjs}}\;o_{\text{ptjs}},\;\phi_{\mathrm{rjs}}\right).$$

The precision parameter ϕ was fitted separately per stage, species, and survey. We allowed for different levels of ϕ to account for the different sampling protocols or sampling areas of the stages. Species‐specific ϕ within stage were necessary because *others*—consisting of multiple species—are assumed to have a much lower dispersion in forests than the stochastic counts of a singular species—*F. sylvatica*. For the initial state, which was constrained with priors based on the first survey in the data, a separate level for ϕ than for the later observations was assumed. Since the sampling areas of size class J changed between surveys, we assumed separate levels for ϕ for all three surveys for this size class, so that overall there were six levels of ϕ in stage J, and each four levels for stage A and B. A very vague half‐normal prior for the inverse of ϕ was specified to improve convergence: $\left(1/\phi \right)∼ℋ\left(10\right)$.

### 2.5 Posterior simulations

#### 2.5.1 Simulating competitive equilibria

**FIGURE 4:**
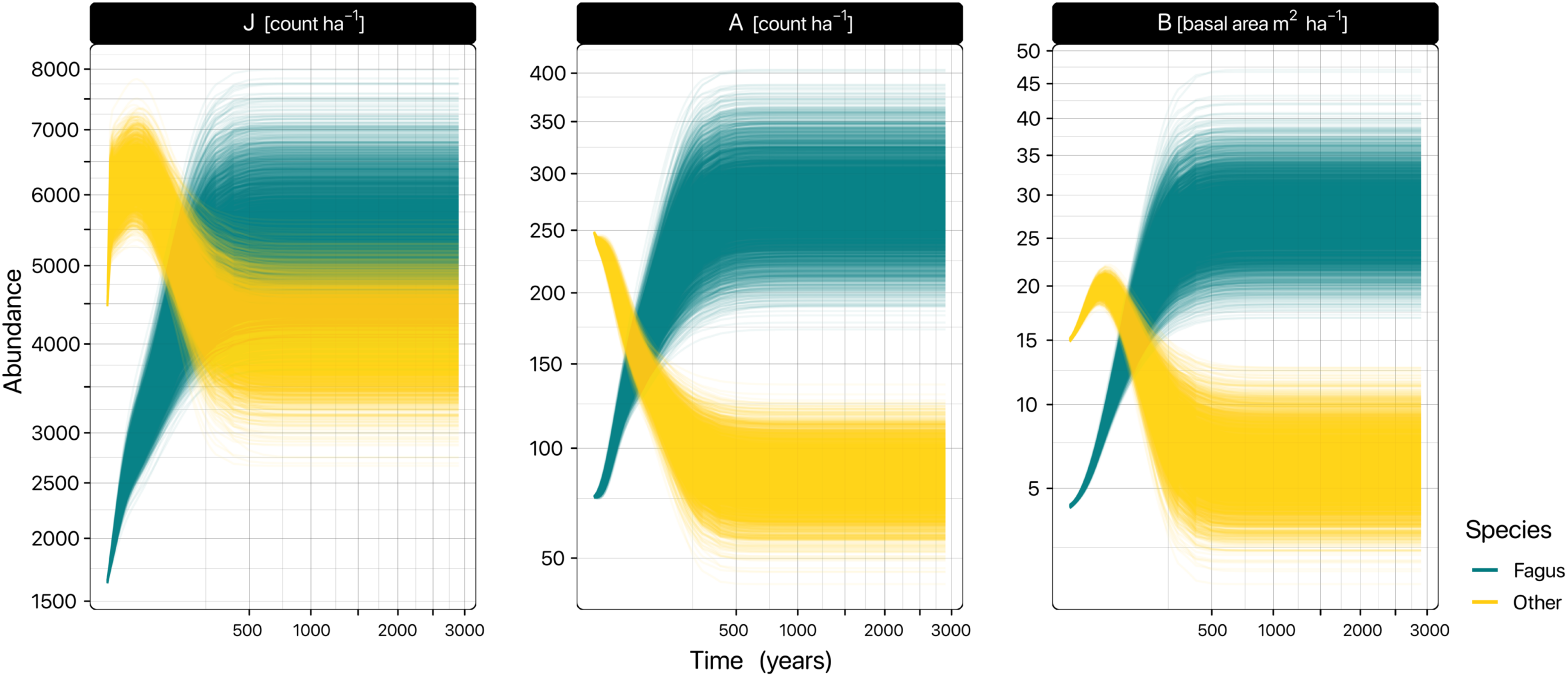
Timeline of stage abundances, simulated forward from the posterior distribution of mean initial states across subpopulations to the equilibrium. The simulations were conducted with the posterior distributions of the parameters, and with the mean per HMC sample of the subpopulation‐dependent seedling input *L*_p_. Hence, the distribution of trajectories reflects both the uncertainty of the initial states per stage, and the parameter uncertainty. Both axes are √‐transformed.

**FIGURE 5:**
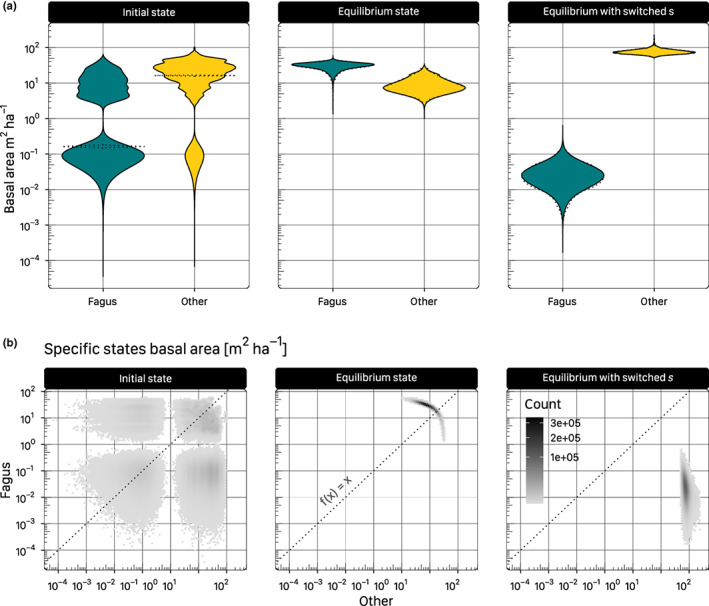
Distributions of basal area of *Fagus* and *others* at the initial state and at the equilibrium as predicted by the JAB model. In addition to the equilibrium simulated with the posterior distribution of the parameters, a counterfactual equilibrium is shown, simulated with the posteriors of the “shading” parameter *s* switched between *Fagus* and *others*. The state distributions include both model uncertainty, as well as spatial variability across subpopulations (NFI plots). (a) While the distribution of initial states of *Fagus* is mostly smaller than that of *others*, *Fagus* has the majority at the competitive equilibrium. The dashed violins represent the model uncertainty of the median initial state across subpopulations. The identical set of subpopulations that represent the median at initial state have equilibrium states spread along the entire range of possible states. (b) Predicted basal area of *Fagus* versus *others* at initial and equilibrium state. The coordinate system is divided into hexagonal bins that are colored by counts of points to indicate density. Points above the diagonal ($f\left(x\right)=x$) indicate *Fagus* predominance, points below indicate predominance of *others*. Note that all continuous axes are log_10_‐transformed.

Using the posterior probability distributions of the parameters, we simulated the JAB model forward from the initial state to obtain trajectories of model states over time (Figure 4) until final equilibrium states were reached (Figure 5). The criterion for reaching equilibrium was that the greatest species‐specific relative change in basal area (BA) in the last time step should not be >1‰: $\max|\left({\mathrm{BA}}_{t}-{\mathrm{BA}}_{t-1}\right)⊘{\mathrm{BA}}_{t}|\leq 0.001$. This numerical method for finding the equilibrium points of the JAB models is equivalent to the established procedure for numerical solution of fixed‐points of a function by formulating the function as an iteration rule (Burden & Faires, 1993). In addition to the equilibrium criterion, the model was simulated over a period of at least 250 years to exclude that potential temporary extrema at the beginning of the trajectory were mistaken as an equilibrium (Figure 4), and for at most 5000 years.

#### 2.5.2 Simulating counterfactual equilibria

**FIGURE 6:**
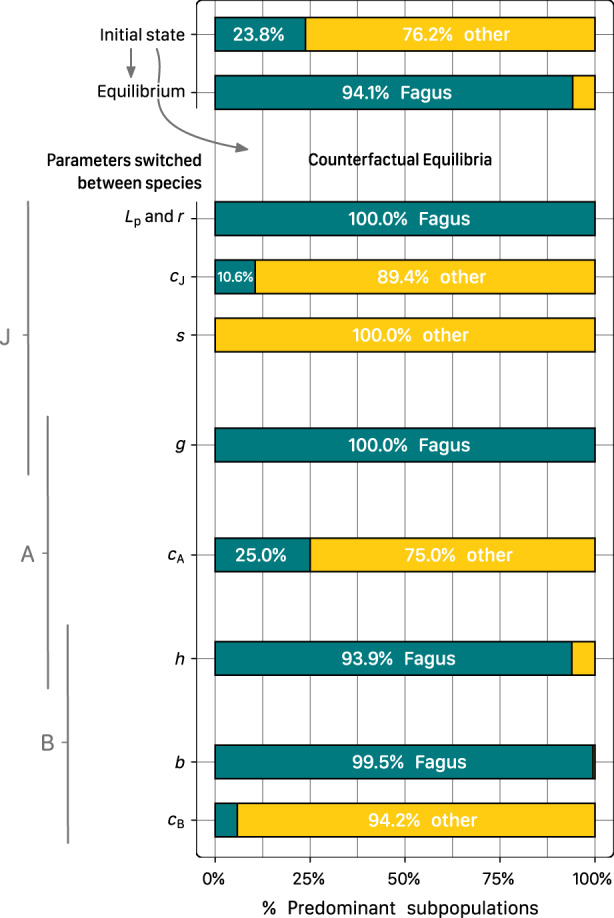
Fraction of posterior cases where either *Fagus* or *others* are the predominant species, at the initial state, at the extrapolated equilibrium, and at counterfactual equilibria with parameters switched between species. Posterior cases include the variance across subpopulations and HMC samples. The seedling recruitment parameters *L*_p_ and *r* were switched jointly.

To test the role of species‐specific differences in the JAB model parameters for the equilibrium, we further simulated new equilibria from the initial state with the parameters switched between species. Additionally, some sets of parameters whose interactions determined demographic processes were switched jointly. Jointly switched parameters include the two seedling recruitment parameters *r* and *l*, as well as parameters that appear together in a density‐dependent term, that is, *g* and *s*, and *c*_J_, *h*, and *c*_A_, *b*, and *c*_B_ (Table 3). By assigning the parameter of *Fagus* to *others* and vice versa in each model run, we generated a distribution of counterfactual equilibria for each parameter and combinations to test how their species‐specific differences drive competitive equilibria (Figure S4; Table 3). As a measure for the role of species‐specific parameter differences in determining the predominance at the equilibrium, we calculated the percentage of cases across subpopulations and HMC samples, that is, “posterior cases,” where either species had the majority after switching the parameters (Figure 6).

### 2.6 HMC sampling

The models, including the JAB model and the model for prior inference on seedling recruitment, were fitted with the Hamiltonian Monte Carlo (HMC) algorithm implemented by the software stan (version 2.21.0; Stan Development Team, 2022). We used the default setting of 1000 iterations for both the warmup and the sampling phase, in four independent HMC chains, so that there were 4000 samples of the posterior distributions (see Table 2 for bulk effective sample size, Vehtari et al., 2021). Convergence of the four chains was checked with stan's default diagnostic $\hat{R}$.

## 3 RESULTS

The JAB model was successfully fitted to size class abundance data from the German NFI (HMC chain convergence for all parameters: $\hat{R}<1.05$), conditioned on prior parameter distributions. Fitting the model with priors to short‐term time series data, we obtained species‐specific demographic rates that were used to extrapolate long‐term trajectories and competitive equilibria of subpopulations (Figure 4). *Fagus sylvatica* was extrapolated to be the predominant species at the competitive equilibrium in about 94% of the posterior cases, despite being in the minority at the initial state. *Fagus*' predominance at the competitive equilibrium was at least partially explained by its weaker response to competition from the overstory compared to *other* species (“shading” parameter *s*), as revealed by the fact that extrapolating new equilibria with *s* switched between species led to *others* predominating in 100% of the cases.

### 3.1 Estimates of demographic rates

We obtained distinct posterior estimates of demographic rates for the two species, despite all rates but *r* having common prior uncertainties (Figure 3, Table 2).

**TABLE 2:** Prior and posterior distributions of JAB model parameters (mean ± standard deviation).

	Posterior		Prior		Bulk ESS	
	Fagus	Others	Fagus	Others	Fagus	Others
log *l*	4.018 ± 0.1156	5.398 *±* 0.1276	4 *±* 2	(common)	4149.079	3494.801
log *r*	2.349 *±* 0.1981	4.825 *±* 0.1296	4.237 *±* 0.5114	3.054 *±* 0.7887	5115.51	4095.912
log *c*_J_	−11.56 *±* 0.1798	−9.770 *±* 0.9153	−10 *±* 2	(common)	3594.346	2055.221
log *s*	−8.908 *±* 1.094	−1.211 *±* 0.5917	−6 *±* 2	(common)	4641.564	1892.538
log *g*	−5.519 *±* 0.06480	−3.591 *±* 0.5471	−5 *±* 2	(common)	4811.408	1923.476
log *c*_A_	−9.421 *±* 1.067	−6.811 *±* 0.2345	−7 *±* 2	(common)	4019.09	4705.809
log *h*	−2.683 *±* 0.06066	−2.843 *±* 0.06431	−4 *±* 2	(common)	5840.295	3412.199
log *b*	−4.779 *±* 0.5712	−3.599 *±* 0.2811	−3.2 *±* 1	(common)	3655.673	2673.14
log *c*_B_	−7.287 *±* 0.2391	−6.691 *±* 0.1724	−7 *±* 2	(common)	3578.249	2760.259
log *g* d.d. init.	−5.582 *±* 0.06156	−5.766 *±* 0.09560	.	.	5065.781	4749.521
log *g* d.d. eq.	−5.615 *±* 0.06115	−6.188 *±* 0.1124	.	.	5111.27	4339.732
log *h* d.d. init.	−2.686 *±* 0.06120	−2.870 *±* 0.06674	.	.	5896.932	3423.927
log *h* d.d. eq.	−2.688 *±* 0.06163	−2.886 *±* 0.06817	.	.	5894.77	3451.546
log *b* d.d. init.	−4.796 *±* 0.5679	−3.629 *±* 0.2763	.	.	3659.276	2672.822
log *b* d.d. eq.	−4.806 *±* 0.5663	−3.647 *±* 0.2740	.	.	3658.505	2669.801
ϕ (J Initial state)	13,810. *±* 107,000.	25,120 *±* 255,000	.	.	3418.828	4024.314
ϕ (J 2nd survey)	0.2561 *±* 0.01868	0.2334 *±* 0.01332	.	.	6993.445	6555.684
ϕ (J 3rd survey)	0.3029 *±* 0.01842	0.2045 *±* 0.01091	.	.	8347.978	7462.385
ϕ (A Initial state)	7896. *±* 166,700.	8034 *±* 58,090	.	.	3156.184	3574.011
ϕ (B Initial state)	9504. *±* 74,570.	16,860 *±* 187,600	.	.	2938.323	4433.032
ϕ (A 2nd/3d survey)	2.047 *±* 0.2750	1.472 *±* 0.1203	.	.	7297.655	8996.458
ϕ (B 2nd/3d survey)	2.399 *±* 0.2246	2.745 *±* 0.1605	.	.	4900.993	7813.761

*Note*: In addition to the growth rates *g*, *h*, and *b*, the corresponding density‐dependent (d.d.) terms are given at initial (init.) and equilibrium (eq.) state, that is, $\log\left\{g⊘\left[1+c_{J}\;\mathrm{sum}\left(J_{t}\right)+s\;\mathrm{sum}\left({\mathrm{BA}}_{t}\right)\right]\right\}$, $\log\left\{h⊘\left[1+c_{A}\;\mathrm{sum}\left({\mathrm{BA}}_{t}\right)\right]\right\}$, and $\log\left\{b⊘\left[1+c_{B}\;\mathrm{sum}\left({\mathrm{BA}}_{t}\right)\right]\right\}$. The dispersion parameter ϕ relates to different levels of uncertainty per stage and survey.

Of the marginal posteriors with common priors for both species (*l*, *g*, *s*, *c*_J_, *h*, *c*_A_, *b*, and *c*_B_), external seedling input *l* and shading‐response *s* were the most differentiated between species (Figure 3). Both *l* and *s* were orders of magnitude higher for *others*. The competition response within J (*c*_J_) was however greater for *Fagus* than for *others*. The overstory parameters (*c*_A_, *h*, *b*, and *c*_B_) were consistently more similar between species with at least some overlap of the posteriors.

The species' sapling transition rates were reversed between conditions without competition (*g*) and with competition (density‐dependent transition term $\log\left\{g⊘\left[1+c_{J}\;\mathrm{sum}\left(J_{t}\right)+s\;\mathrm{sum}\left({\mathrm{BA}}_{t}\right)\right]\right\}$; Figure S3). While transition without competition *g* was lower for *Fagus*, the density‐dependent transition term was much higher than that of *others*, especially at the competitive equilibrium. The interspecific differences of demographic rates *h* and *b* were, however, mostly unaffected by density effects (Figure S3; Table 2).

The marginal parameter estimates for seedling recruitment *r* deviated from their species‐specific priors (Figure 3; Table 2). The internal seedling recruitment rate *r* was smaller than the prior for *Fagus* and greater for *others*, which had much greater posterior values, overall.

A multivariate representation of the posterior parameter distributions revealed strong correlations among sets of certain parameters (Figure S2): In particular, *others*' *g* was strongly correlated with its response to competition *s* and *c*_J_ (Pearson correlation .98 and .90, respectively). In addition, there were strong correlations between the two parameters determining the basal area growth in *B*, *b*, and *c*_B_, of both species, *Fagus* (.83) and *others* (.96).

### 3.2 Initial state and extrapolated competitive equilibria

All subpopulations across all HMC samples reached a competitive equilibrium (Section 2.5.1) after forward simulation over a median of 381 and a maximum of 1169 years (Figure 4).

At the initial state, *Fagus* was the less common species, only predominant in 24.0% of the cases across subpopulations and HMC samples—predominance being defined as constituting >50% of the modeled basal area (Figure 6, Table 3). Also, the mean basal area of *Fagus* was much lower at 5.1 m^2^ ha^−1^ than *others*, which predominated at mean 18.4 m^2^ ha^−1^ (Figure 5; Table 3). In general, the average predicted initial model states were close to the average initial observations in the data but slightly larger (Table 3).

**TABLE 3:** Posterior distributions of initial and equilibrium abundances (stages J and A [count ha^−1^]; B and total BA [m^2^ ha^−1^]) and species' predominance (mean ± standard deviation across subpopulations and HMC samples).

		Fagus	Others
J	Initial state data	1623.4 *±* 5361.1	4467.9 *±* 13,412.
	Initial model state	1649.3 *±* 5357.6	4492.1 *±* 13,406.
	Equilibrium state	5265.2 *±* 1331.1	4304.5 *±* 1272.1
A	Initial state data	72.964 *±* 209.83	245.78 *±* 453.27
	Initial model state	75.714 *±* 208.94	247.92 *±* 452.14
	Equilibrium state	262.55 *±* 67.135	92.517 *±* 33.499
B	Initial state data	4.0505 *±* 7.5868	14.872 *±* 15.390
	Initial model state	4.1454 *±* 7.6761	15.016 *±* 15.509
	Equilibrium state	25.971 *±* 6.1065	7.5556 *±* 3.9755
BA	Initial state data	5.1049 *±* 8.5159	18.395 *±* 16.071
	Initial model state	5.3459 *±* 8.6968	19.010 *±* 16.354
	Equilibrium state	30.133 *±* 7.0646	9.0462 *±* 4.4942
	Eq. Sum across species	39.179 *±* 3.0684	
	Eq. With switched *l* and *r*	84.681 *±* 9.7323	0.048748 *±* 0.028346
	Eq. With switched *c*_J_	7.1219 *±* 8.4019	25.682 *±* 6.4551
	Eq. With switched *s*	0.030156 *±* 0.025767	76.468 *±* 12.579
	Eq. With switched *g*	131.84 *±* 69.280	0.11712 *±* 0.17244
	Eq. With switched *g*, *c*_J_, and *s*	0.32823 *±* 0.17863	52.894 *±* 4.0761
	Eq. With switched *g* and *s*	1.0286 *±* 0.85662	36.204 *±* 7.6819
	Eq. With switched *c*_A_	14.715 *±* 6.1191	22.331 *±* 5.6985
	Eq. With switched *h*	30.229 *±* 7.1389	9.2249 *±* 4.5736
	Eq. With switched *h* and *c*_A_	14.086 *±* 6.0263	22.691 *±* 5.6290
	Eq. With switched *b*	58.235 *±* 15.821	2.2481 *±* 2.6158
	Eq. With switched *c*_B_	7.4086 *±* 6.1813	43.081 *±* 16.073
	Eq. With switched *b* and *c*_B_	26.032 *±* 6.2534	11.856 *±* 5.1306
Frequency of predominance	Initial model state	0.23812 *±* 0.42593	0.76188 *±* 0.42593
	Equilibrium state	0.94142 *±* 0.23484	0.058581 *±* 0.23484
	Eq. With switched *l* and *r*	1.0000 *±* 0	0 *±* 0
	Eq. With switched *c*_J_	0.10564 *±* 0.30738	0.89436 *±* 0.30738
	Eq. With switched *s*	0 *±* 0	1.0000 *±* 0
	Eq. With switched *g*	1.0000 *±* 0	0 *±* 0
	Eq. With switched *g*, *c*_J_, and *s*	0 *±* 0	1.0000 *±* 0
	Eq. With switched *g* and *s*	0.00022925 *±* 0.015139	0.99977 *±* 0.015139
	Eq. With switched *c*_A_	0.25013 *±* 0.43309	0.74987 *±* 0.43309
	Eq. With switched *h*	0.93882 *±* 0.23966	0.061177 *±* 0.23966
	Eq. With switched *h* and *c*_A_	0.22204 *±* 0.41562	0.77796 *±* 0.41562
	Eq. With switched *b*	0.99471 *±* 0.072535	0.0052892 *±* 0.072535
	Eq. With switched *c*_B_	0.058321 *±* 0.23435	0.94168 *±* 0.23435
	Eq. With switched *b* and *c*_B_	0.87634 *±* 0.32919	0.12366 *±* 0.32919

*Note*: Equilibrium states include counterfactual simulations, where some parameters have been switched between *Fagus* and *others*.

Although initially, *Fagus* was in the minority, it became predominant in around 94% of the posterior cases at the competitive equilibrium (Figure 6; Table 3). At equilibrium, *Fagus* also superseded *others* in terms of basal area (mean 30.1 and 9.0 m^2^ ha^−1^, respectively; Figure 5; Table 3). High equilibrial basal areas of *Fagus* were always correlated with low basal areas of *others*, and vice versa (Figure 5b). Further, focusing on the distribution of the subpopulations that represented the median across HMC samples at the initial state revealed that these subpopulations, at the equilibrium state had the same distribution as the total of all subpopulations (Figure 5a).

### 3.3 Counterfactual equilibria with switched parameters

To test the role of species differences in demographic processes for determining the competitive equilibrium, we simulated counterfactual equilibria with the parameter values switched between species (Figure 6; Table 3).

Compared to the original equilibria, where *Fagus* was extrapolated to predominate in 94% of the cases across subpopulations and HMC samples, only switching one of the limiting parameters *c*_J_, *s*, *c*_A_, and *c*_B_ led to a major change in the distribution of basal area across species and in the frequency of predominance (Table 3; Figure S4). In particular, switching the posterior estimates of *s* between species had the most pronounced effect (Figure 5), leading to predominance of *others* in 100.0% of the cases, that is, reversing the predominance in all of the cases where Fagus would have predominated before. The limiting parameter in B, *c*_B_, had the second greatest effect on predominance at the equilibria, leading *others* to predominate in 94.2% of the cases. Switching of *L*_p_, *r*, *g*, and *b*, only amplified Fagus' predominance.

Switching the density‐dependent rates *g*, *h*, and *b* together with their limiting counterparts so that the results refer to the species‐specific differences in the density‐dependent transitions and growth together with the density effect on the states, led to a major change in predominance for the joint switch of *g* and *c*_J_, and *s*, as well as for *h* and *c*_A_ (Figure S5). The counterfactual equilibrium after joint switch of *b* and *c*_B_, however, had a similar outcome as the extrapolated equilibrium (87.6% *Fagus*).

## 4 DISCUSSION

Here, we inferred species‐specific demographic rates of *Fagus sylvatica* and all *other* species by fitting the JAB model to short‐term time series data from the German NFI with prior parameter distributions for seedling recruitment based on external data from the Slovakian NFI. Using the posterior estimates of the demographic rates, we extrapolated long‐term population trajectories. Consistent with the traditional consensus, *Fagus* was extrapolated to predominate across competitive equilibria, despite being in the minority at the initial state. The most important demographic process in sustaining *Fagus*' predominance is the weaker competition response of its saplings to the overstory, as demonstrated by simulations of counterfactual equilibria.

### 4.1 Estimates of demographic rates

The demographic rates, which were estimated globally across all subpopulations and different environments, revealed species‐specific differences between *Fagus* and *others* (Table 2) that largely followed expectations from demographic traits at different positions along a pioneer—late‐successional spectrum (Grime, 2001; Whitmore, 1989), especially in the sapling stage J.

Saplings of *Fagus*, consistent with the consensus on their shade tolerance (Ellenberg, 1963; Petrovska, Brang, et al., 2021; Watt, 1923), were less affected by competition from the overstory (*s*) than saplings of the *other* species. Although the estimated effect of *s* also includes belowground competition (Coomes & Grubb, 2000; Putz & Canham, 1992), we assume the response to shading to be the most important component of the competition response of saplings to the overstory. The competition response of J to itself (*c*_J_) was orders of magnitude smaller for *others*, even when considering the difference of units between J and BA (Table 3), which indicates that the density‐dependent limitation of *others*' saplings is mainly caused by the overstory. That *others*' seedling recruitment parameters *l* and *r* had considerably higher estimates, is expected from a species mixture including pioneer species (Table S3). Especially, the external seedling input *l* is expected to be much lower in *Fagus sylvatica* because, compared to other tree species, it only rarely disperses over larger distances (Dounavi et al., 2010; Kunstler et al., 2007). In comparison to these parameters, the transition rate *g*, that is, growth from stage J to A in the absence of competition, was less differentiated between *Fagus* and *others*. The overstory‐related parameters (*c*_A_, *h*, *b*, and *c*_B_) were overall more similar among species, which suggests that a major part of the demographic differentiation happens in the sapling stage J. Still, the net basal area increment *b* of *Fagus* was relatively slower compared to the *other* tree species, whose basal area in the German NFI mostly consists of fast‐growing timber species (Vospernik, 2021; Table S3).

The parameter estimates have to be interpreted in the context of two major trade‐offs in the model structure, which were revealed by correlations between the posteriors of *others*' sapling parameters *g*, *c*_J_, and *s*, as well as in the overstory between *others*' *b* and *c*_B_ (Figure S2). These strong correlations indicate a certain degree of equifinality, that is, that the same model states that fit the data can be produced by multiple parameter configurations (Hartig et al., 2014), for example, the same high basal area could be produced by either fast growth or a small competition effect. Consequently, the parameters *g*, *c*_J_, and *s*, as well as *b* and *c*_B_ should be interpreted in conjunction to understand their interaction, for example, by interpreting the complete density‐dependent transition term $\log\left\{g⊘\left[1+c_{J}\;\mathrm{sum}\left(J_{t}\right)+s\;\mathrm{sum}\left({\mathrm{BA}}_{t}\right)\right]\right\}$ (Table 2) and by jointly switching parameters between species to generate counterfactual equilibria (Section 4.3).

In contrast to the parameter estimate of *g*, the density‐dependent transition term at the sapling stage $\log\left\{g⊘\left[1+c_{J}\;\mathrm{sum}\left(J_{t}\right)+s\;\mathrm{sum}\left({\mathrm{BA}}_{t}\right)\right]\right\}$ was distinctly greater for *Fagus*. The density‐dependent growth and survival terms in the overstory, however, were not fundamentally different than the density‐independent rates *h* and *b* (Table 2, Figure S3). This hints at the exceptional role of competition effects on growth and survival at the sapling stage.

That the posterior estimates of seedling recruitment *r* deviated from the species‐specific priors, indicating a conflict between the prior external information and the aggregated population data that the JAB model is fitted to, can be explained by possible biases in the estimation method, which assumes that density of small seedlings are a species‐unspecific proxy for yearly seedling input (see Appendix A.4). Furthermore, the differences in geographical conditions and silvicultural history in Slovakia might have biased the estimates.

Overall, the population dynamics as suggested by the demographic rates of *Fagus* compared to *others* can be subsumed as slower population growth through seedling input (*l*, *r*) but overall higher sapling survival (joint effect of *g*, *c*_J_, *s*), and predominance despite slower net basal area growth (interaction of *b* and *c*_b_), which is consistent with the demographic strategy described by Petrovska, Brang, et al. (2021) as: “grow slowly, persist, dominate.”

### 4.2 Predominance at the competitive equilibrium

*Fagus* was extrapolated to be the predominant species in 94% of the competitive equilibria although being in the minority at the initial state, that is, the state corresponding to the first of the German NFI surveys. This long‐term extrapolation of population trajectories was based on short‐term demographic rates that were estimated globally, that is, each JAB model parameter integrated all subpopulations without being allowed to vary systematically with the environment. According to the traditional consensus, *Fagus* is very competitive under mesophilic conditions but relatively intolerant to abiotic stress like drought on the one hand, or anoxia on the other hand (Ellenberg, 1963; Ferner et al., 2012; Leuschner, 2020; Meier et al., 2011). Under the assumption that physiological limitation under extreme conditions is mostly independent of species interactions, it follows that the selected plots where *Fagus* was observed at all (see Appendix A.1.1), do likely not only represent the environmental range where Fagus is able to physiologically persist but at the same time the range where it is competitive. Consequently, it is expected that *Fagus*' is also predominant in the majority of equilibria that were extrapolated from globally inferred estimates of demographic rates on these plots.

The projected total equilibrium basal area of around 39 m^2^ ha^−1^ (Table 3) lies between the total basal area reported from pristine beech‐dominated forests (~36 m^2^ ha^−1^ in Uholka‐Shyrokyi Luh, Ukraine; Stillhard et al., 2022) and the upper limits of European forests around 50 m^2^ ha^−1^ projected by Moreno et al. (2018). A maximum carrying capacity at the combined basal area below 50 m^2^ ha^−1^ is also indicated by the line formed by bivarietely associated basal areas of *Fagus*' and *others*' (Figure 5b, center).

Uncertainty of the equilibrium states originates mostly from the posterior uncertainty of the model parameters. This is demonstrated by visualizing the equilibrium distribution for a single subpopulation (taking a population at the median of the initial state, Figure 5). Given that this single subpopulation had the same uncertainty as the total distribution of subpopulations indicates that the equilibrium is independent of the subpopulation‐specific initial state and regional basal area $B_{p}$. In conclusion, instead of the subpopulation states, the parameter uncertainty is responsible for most of the variation in the equilibria.

Comparing the means of the initial model states and the corresponding stages in the data reveals small but consistent overestimation across size classes (Table 3), which can be explained by the model assumptions for the latent model states. The initial model state was fitted to the data as a continuous, strictly positive random variable with strong priors around the data, which were less certain for observed zeroes (Section 2.4.1), so that the predicted estimates are distributed more densely around the count levels, including many values close to but not zero (Figure 5a). Nevertheless, this approach allowed the initial states to flexibly adapt to the priors on the one hand and to the model structure with transitions between stages on the other hand, so that continuous states that had corresponding zeroes in the data were likely pulled up to higher values if the priors or the other model stages made it likely.

In general, the predicted model states of the pooled species *others* are to be interpreted as an approximate environmental variable that affects the *Fagus* population, rather than a population itself. For this analysis with the focus on *Fagus*, multiple species have been aggregated into one, also resulting in a pooled estimate of the different demographic properties within the pool. This also assumes that the parameters of *others* are constant over the succession. In reality the species composition within *others* would probably change with succession towards more competitors in the pool (Lortie et al., 2004; Vellend, 2010), for example, *Abies alba* in colder regions (Leuschner & Ellenberg, 2017), and consequently change the overall demographic properties of *others* towards more competitiveness. On the other hand, while the model assumes constant regional basal area $B_{p}$ that informs the seedling input into the subpopulation, in reality, the regional basal area would shift toward the predominant species, which would provide *Fagus* a dispersal advantage that is not reflected in the model.

### 4.3 Which species‐specific differences in demographic rates lead to predominance?

We tested the hypothesis that the weaker shading effect on saplings plays an important role in sustaining *Fagus*' predominance at the competitive equilibrium (Petrovska, Brang, et al., 2021; Watt, 1923) with counterfactual simulations of equilibria where parameters had been switched between species (Table 3; Figure S4). The results clearly confirm the key role of the shading parameter *s*, which reversed all equilibria to the benefit of *others* when switched (Figure 6). Species differences in the tolerance to overstory competition also played an important role for *Fagus*' predominance after the sapling stage, as evident from the high predominance of others after the switch of *c*_A_ and *c*_B_. The species differences in growth parameters *g*, *h*, and *b* in the absence of competition, are all beneficial to *others*' equilibria, as indicated by *Fagus*' even greater predominance after the switch. The seedling recruitment parameters *L*_p_ and r were switched jointly because they are part of a similar process and both posteriors are beneficial for *others*, but they are trumped by the greater limiting effect of *s* and *c*_J_ on others.

The shading parameter *s* also had the most important role in counterfactual equilibria after joint switches of the rates *g*, *h*, and *b* with their limiting counterparts (Figure S5). When *s* is switched together with *g*, *others* still predominate in 100% of the cases, even though the switched *g*, should improve *Fagus*' dynamics. In contrast, the strong positive effect of the shift of *c*_B_ on the abundance of *others*, disappeared when *c*_B_ and *b* were shifted jointly. Thus, when the correlation between *c*_B_ and *b* is accounted for, the joint effect of the overstory parameters does not appear to be a key determinant of *Fagus*' predominance. That the switch of *s* reversed all projected equilibria to the benefit of *others* is, however, not challenged by the joint switches with correlated parameters.

Overall, the demographic differentiation in the sapling stage, particularly the weaker response of *Fagus* to shading (*s*), is responsible for sustaining the predominance at the competitive equilibrium, which is in line with the traditional view of shade tolerance leading to long‐term predominance (Ellenberg, 1963; Watt, 1923). Furthermore, our findings are consistent with the observation that for the recruitment success and predominance of *Fagus*, survival of saplings under shading, that is, a smaller competitive response *s* to the overstory, is more important than increased growth rates (Kunstler et al., 2005; Petritan et al., 2007; Petrovska, Bugmann, et al., 2021; for this mechanism in other shade‐tolerant species see also Canham et al., 1999; Kitajima, 1994; Kobe et al., 1995).

### 4.4 Prospects for the JAB model

Having demonstrated the ability of the JAB model to be fitted to forest inventory data available at large spatial scales, and used for inference about the demographic processes underlying species predominance, there are multiple possible extensions of the model that could be applied in future research.

Formulated in the modeling language stan, the JAB model can be easily extended with several structural changes: (1) spatial random effects structure for the parameters, (2) temporal random effects structure for the parameters (e.g., adding random pulses to the seedling input), or (3) temporal process errors for transitions between the model states. These different structural extensions are ways to further clarify the parameter estimates by allocating some of the current variability in the parameters to the sampling structure (Section 4.2).

Further, the JAB model will efficiently scale up to multiple species. Calculations within the stan implementation are primitive vector operations from the software library Eigen that scale up efficiently (Guennebaud & Jacob, 2010; Stan Development Team, 2022). The number of estimated parameters will scale linearly with the number of species, because we reduced the interactions compared to a traditional Lotka–Volterra model to have only one‐directional competitive effects (Section 2.1).

Furthermore, species abundances along environmental gradients could be explained by linking the demographic processes in the JAB model to environmental predictors at the subpopulation level (Briscoe et al., 2019; see also Schultz et al., 2022). This way, questions like “How does the variation in *Fagus*' tolerance to shading at the sapling stage (parameter *s*) explain its variation in predominance along environmental gradients?” are readily answered with the posterior simulations that we demonstrated in Section 2.5.

## 5 CONCLUSIONS

Here, we proposed the JAB model, a parsimonious multi‐species system of size‐structured tree populations that concentrates on the interactions between saplings in the understory and adult trees in the overstory. We demonstrated the JAB model's ability to extrapolate credible long‐term competitive equilibria from a fit to short‐term time series data (see Damgaard, 2019). Using commonly available data from the German NFI with external information from the Slovakian NFI allowed us to extrapolate that the competitive, shade‐tolerant species *Fagus sylvatica* will be predominant at the competitive equilibrium in a two‐species system of *Fagus* and all *other* tree species, although *Fagus* has been in the minority initially.

We further used the posterior parameter distributions from the JAB model fit to demonstrate with simulations that the species‐specific differences in demographic processes of the sapling stage are crucial in determining the competitive equilibrium between tree species. In particular, we found strong evidence for the hypothesis that the higher tolerance of *Fagus* saplings to shading and other competition from the overstory is key for long‐term predominance of *Fagus*. Our findings highlight the proposition that limitations in early ontogenetic stages constrain the potential outcomes at later stages and, in particular, the crucial role of the seedling and sapling stage for species assembly in forest communities (Grubb, 1977; Heiland et al., 2022; Maguire, 1973; Young et al., 2005).

Finally, the JAB model presented here can be extended to be applied in future research, like using systems of multiple species and linking the demographic processes to the environment to build demographic distribution models. The JAB model could be instrumental in improving our understanding of how demographic processes at the sapling stage control forest composition of tree species communities across the environment.

## AUTHOR CONTRIBUTIONS

**Lukas Heiland:** Conceptualization (lead); data curation (lead); formal analysis (equal); methodology (lead); software (equal); writing – original draft (equal); writing – review and editing (equal). **Georges Kunstler:** Conceptualization (equal); formal analysis (equal); methodology (supporting); supervision (equal); writing – review and editing (equal). **Vladimír Šebeň:** Data curation (equal); writing – review and editing (supporting). **Lisa Hülsmann:** Conceptualization (equal); formal analysis (equal); methodology (supporting); project administration (equal); supervision (lead); writing – review and editing (equal).

## CONFLICT OF INTEREST STATEMENT

No competing interests.

## Supporting information


Appendices A–E

## Data Availability

The data supporting the findings of this study have been made available by the authors at https://doi.org/10.5061/dryad.3ffbg79pv. All software to reproduce the study, including the JAB model, has been made openly available at https://doi.org/10.5281/zenodo.8032461.
