# Supplementary material for: Which demographic processes control competitive equilibria? Bayesian calibration of a size‐structured forest population model
Source: Ecol Evol. 2023 Jul 4;13(7):e10232. doi: 10.1002/ece3.10232 (PMC10318622; doi:10.1002/ece3.10232)
Supplement: Supplementary file 1 — Appendices A–E [file ECE3-13-e10232-s001.pdf]

Appendices to

Which demographic processes control competitive equilibria? Bayesian calibration of a size-structured forest population model

Lukas Heiland (corresponding author), University of Bayreuth, Bayreuth Center of Ecology and Environmental Research (BayCEER), Ecosystem Analysis and Simulation (EASI) Lab, Dr.-Hans-Frisch-Str. 1–3, 95448 Bayreuth, Germany. University of Regensburg, Theoretical Ecology, Universitätsstraße 31, 93053 Regensburg, Germany. ecology@heiland.earth. ORCiD: https://orcid.org/0000-0002-0570-2138.

Georges Kunstler, Université Grenoble Alpes, Inrae, LESSEM, 38000 Grenoble, France. georges.kunstler@inrae.fr. ORCiD: https://orcid.org/0000-0002-2544-1940.

Vladimír Šebeň, Národné lesnícke centrum, T. G. Masaryka 2175/22, 96001 Zvolen, Slovakia. vladimir.seben@nlcsk.org. ORCiD: https://orcid.org/0000-0003-3692-446X.

Lisa Hülsmann, University of Bayreuth, Bayreuth Center of Ecology and Environmental Research (BayCEER), Ecosystem Analysis and Simulation (EASI) Lab, Dr.-Hans-Frisch-Str. 1–3, 95448 Bayreuth, Germany. lisa.huelsmann@uni-bayreuth.de. ORCiD: https://orcid.org/0000-0003-4252-2715.

Appendix A Methods

Appendix A.1 NFI data

Appendix A.1.1 German NFI data

Germany is situated in the European temperate vegetation zone, with the main potential natural vegetation being beech (*F. sylvatica*) and mixed beech forests (Bohn and Gollub, 2006). The NFI plots used for calibration, which have been selected to include at least some observation of *Fagus*, have *Fagus* as the most abundant species with an average of about 24% of the basal area (Table S3).

The forest plots of the German NFI are arranged in clusters of four plots on a regular quadratic grid. The standard grid has clusters arranged at longitudinal and latitudinal distance of 4 km. In some federal states the grid may be intensified to either the grid centers (distance 2.83 km) or the sides (distance 2 km) of the grid quadrants (Tomppo et al., 2010). Each grid point is the position of the south-western plot within a quadratic cluster of four potential forest plots, which are arranged at distance 150 m. To fit the JAB model, we used one randomly selected forested plot of a cluster corresponds to a subpopulation. One plot per cluster was selected, instead of averaging or summing up per cluster, because the within-cluster variance of abundances was very high, yet selecting one plot per cluster still avoids potential dependencies among the plots of one cluster.

In the German NFI data, trees above 10cm dbh were sampled with the angle count method (Riedel et al., 2017), which selects all trees that appear wider than a defined angle around a central observer at height 1.3 m (Kangas and Maltamo, 2006). The selected trees are then recorded together with their distance and diameter at breast height (dbh, 1.3 m). Instead of a fixed area for all tree sizes, the selection with an angle leads to a sampling area that varies with dbh. The angle count method—besides providing counts that are directly proportional to the basal area—delivers data where each counted tree has (1) a corresponding distance to the center, (2) a dbh that is equivalent to a potential sampling distance, i.e. area and sampling probability, (3) as well as a calibration factor k [m^2 ha^{-1}] for the sampling angle. In this study, the angle count plots were truncated to have a fixed maximum area of 14 m (for details see Section Appendix A.2.3). Saplings between height 20 cm and dbh 7 cm were counted on circular fixed area plots with radii dependent on size class and survey and were subsumed as size class J in the analyses (Table S2).

For analyzing potential predominance of *Fagus*, we selected plots with repeated observations that were unmanaged during the observation period: First, to rule out clear cuts, we excluded all plots that had tree observations in the first or second of the three surveys, but had only zero-observations across all tree sizes in a subsequent survey. Further, plots where management was recorded in the second or third survey were excluded. Records of management in the German NFI include unnatural forest regeneration (sowing, planting), harvested sample trees, and damage through forestry in sample trees. We also excluded plots with records of individual sample trees that went missing for "unknown reasons" in the third survey (category not available in the first and second survey). Subsequently, to make sure that the data is robust for inferring trajectories of populations over time, plots were selected that were surveyed thrice. Selecting only plots with observations in all three surveys—1987, 2002, and 2012—confines the spatial extent of the German NFI to former West Germany. Further, to make sure that only locations are selected that are likely within the fundamental niche of *Fagus sylvatica*, we limited the data to plots that had any record of *Fagus* (and *others*) in any size stage at any time and to plots at submontane elevations 100–600m (Ellenberg, 1963). From the remaining clusters we sampled one random plot per cluster. Finally, to limit the computational expense, 1000 plots each representing one cluster were randomly sampled (compared to originally 61666 plots in 21574 clusters; Table S1).

Appendix A.1.2 Slovakian NFI data

The forested areas in Slovakia are mostly part of the European temperate vegetation zone and have similar potential natural beech and mixed beech forests (Bohn and Gollub, 2006) and current species composition (Tables S3–S4) as in Germany. The selected forest plots in Slovakia have *F. sylvatica* as the predominant species with about 28% of the basal area.

In the Slovakian NFI, full tree counts and circumference measurements were conducted on 500 m^2 fixed-area plots that are arranged on a regular quadratic grid (Šebeň, 2017). The lower size threshold for sampling trees is dbh 7 cm. Smaller seedlings and saplings with height 10 cm and greater were sampled on varying circles with increasing areas, i.e. 3.14, 7.07, 12.57, 28.27, and 50.26 m^2. The sampling area for seedlings and saplings was adapted depending on field estimates of the tree density so that at least 15 trees were counted.

To infer seedling recruitment rates from the Slovakian NFI, we selected only plots without records of unnatural regeneration. This reduced the number of plots from 1412 to 1402 (Table S1).

Appendix A.2 Accounting for varying sampling areas with offsets

Appendix A.2.1 Offsets for the angle count method

Count data from the angle count method (size stage A in the German NFI) were related to the count states in the JAB model by scaling with area offsets. In angle count sampling, each tree has a specific sampling area a dependent on its dbh with $a = \pi\, k^2\, dbh^2$, where k is a constant for the sampling angle (here, $k = 25$). Thus, for the sampling area of a plot, which corresponds to the actually observed trees on a plot we use the weighted mean $a_p = \sum_{i=1}^{n} w_i a_i \,/\, \sum_{i=1}^{n} w_i$, where the weights w_i are the respective area-standardized counts per hectare. This weighted mean has the property that—together with the actually observed counts on a plot c_p—it conveys equivalent information as the sum of all area-standardized counts per hectare on a plot $c_A = \frac{c_p}{a_p}$ [ha^{-1}]. The area a_p was used as the offset for size stage A (o in Equation **??**).

To relate count data from the angle count method to the basal area state in the model (size stage B in the German NFI), we used an offset factor that not only includes the sampling area a_p but transforms the basal area per hectare in the JAB model to counts. This was achieved with an offset o that also included the total observed basal area ba_p on a plot: $o = a_p \frac{c_p}{ba_p}$ [$ha\,m^{-2}$]. By multiplying the model state $\hat{x}$ [$m^2\,ha^{-1}$] with this offset we transformed the model state to counts, by including the data on basal area ("how many counts are one unit of basal area?") on the right hand side of the model statement: $x \sim\ a_p \frac{c_p}{ba_p}\, \hat{x}$.

Appendix A.2.2 Offsets for counts on fixed-area plots

Counts on fixed-area plots (seedlings in the Slovakian NFI and saplings J in the German NFI) were also standardized using area offsets. In the statistical model inferring priors for the seedling recruitment rates r from the Slovakian NFI data (see Section Appendix A.4), we used areas of the corresponding subplots as offsets. In the German NFI data, the size class J of the JAB model consists of multiple size classes with different sampling areas, which also change between surveys (Table S2). As in the angle count data, we calculated a weighted mean area per plot, where the different sampling areas were weighted by the counts per area within the corresponding size classes. The weighted mean area per NFI plot was used as an offset to relate the sapling counts to the area-standardized counts of J in the JAB model [ha^{-1}].

Appendix A.2.3 Offsets for zero observations

Because all NFI sampling protocols made observation areas dependent on size or abundance (see Section Appendix A.1.2 and Appendix A.1.1), the observation area was not readily available when there were no observations for a stage on a plot. For zero observations in the angle count data in the German NFI (size classes A and B), we used the sampling area of the other species, or, if both species had zero observations, the mean sampling area per size class and survey as an offset (for a test of the robustness of this approach, see below). To be able to compute a mean area for the largest size class B, we truncated the angle count data to a maximum sampling radius of 14 m. The truncation included dropping all trees outside the sampling radius, which is about the 98th percentile of tree counts in A and B and assigning the new maximum sampling area to all trees that were within the maximum radius but had a dbh that was originally corresponding to a sampling area greater than the new maximum area (dbh > 60 cm). Truncating the angle count data to a fixed maximum area removes a source of bias (missed tree observations at higher radii) at the cost of moderate added variance (Berger et al., 2020), and furthermore leads to a finite sampling area that can be averaged over. The mean sampling area per size class (A and B) was calculated by dividing the definite integral from 0 up to the respective maximum radius—by the maximum radius. This mean area was used as an offset for zero observations in size class A. To obtain an offset for zero observations in size class B, we also needed an additional factor that scaled the basal area to the scale of observed counts. Instead of multiplying the area with the plot-specific factor $\frac{c_p}{ba_p}$ (as for non-zero observations, see Section Appendix A.2.1), we included the mean of the factor per survey and additionally per species to differentiate between their average basal areas.

We made sure that the choice of the particular area offset for zero observations did not affect the relations of the parameter estimates, nor the conclusions regarding the extrapolated equilibria, nor the importance of the "shading" parameter s, by running the entire analysis pipeline with three alternative choices for the sampling area of zero observations: the count per hectare-weighted mean of all observation areas per survey and species, as well as the analogous first quantile and the third quantile (weighted quantiles from package DescTools version 0.99.45; Signorell, 2021).

Zero observations in the sapling counts of the size class J in the German NFI were assigned an equally weighted mean of all possible sampling areas for the respective set of smaller size classes within the corresponding survey (Table S2).

For zero observations in seedling counts in the Slovakian NFI there were two possible cases: If only one of *Fagus* and *others* had no seedling observation on a plot in a year, the zero observation was assigned the sampling area from the respective other species. If there was no seedling observation at all, we used the maximum possible area (50.26 m^2), because the sampling protocol prescribed increasing the subplot area until sufficient seedling density was reached (see Section Appendix A.1.2).

Appendix A.3 Species' regional abundance for predicting seedling input

To derive the regional species abundance $\mathfrak{B}_p$ used to estimate the external seedling input (parameter l, see Section **??**) we used thin plate spline regressions with the geographic coordinates as predictors to interpolate the species-specific basal area per hectare on the German NFI grid (Table S6, Figure S1).

For fitting the thin plate splines from the regular standard grid of the German NFI (4 km; see Section Appendix A.1.1), which is homogeneous across Federal states, we selected only the basal area records from the last survey (2012), because the grid had changed over surveys. We completed non-forested clusters of the regular grid, and also all of the four plots within clusters, with zero observations to obtain an unbiased sample of the geographical density of tree species. To obtain a response variable for each set of coordinates, we calculated the

species abundances per cluster by averaging the basal area per hectare above the sampling threshold (BA) over the four plots. This average basal area per hectare on the completed grid was spatially smoothed with a two-dimensional geographic thin plate spline. Based on the coordinates, a "spline on sphere" was fitted with the `gam()` function from the package mgcv (`k = 200`; version 1.8-39; Wood, 2021), where the average basal area per hectare was the response. The response variable had been rounded to an integer to be able to fit a negative binomial response. The predicted plot-specific regional species abundances $\mathfrak{B}_p$ were used as a proxy for the probability of seedling input, which factors into the JAB model with a species-specific effect size l, $L_p = \mathfrak{B}_p\, l$. By using a strictly positive negative binomial response for $\mathfrak{B}_p$, it was made sure that plots were not excluded from invasion in the JAB model.

Appendix A.4 Prior inference on seedling recruitment

To infer priors for the rate r, i.e. seedling recruitment by the local basal area, we fitted a regression model with the data from the Slovakian NFI (Table S5). The Slovakian NFI includes counts of small seedlings with height 10–20 cm, which is right below the size class J in the German NFI (height 20 cm to dbh 7 cm, Section **??**). The density of the small seedlings on a plot was used as a proxy for the number of seedlings having appeared in a year on a plot. Although the size class height 10–20 cm neither completely covers yearlings nor excludes older or younger trees, data on seedling growth suggests that this size class can be a rough approximation for yearling count density: The common conifer *Pinus sylvestris* L., e.g., reaches average height 20.0 cm after two years (Geudens et al., 2004), *Fagus sylvatica* and *Quercus robur* L. both have an average height between 10 and 15 cm after one year (Welander and Ottosson, 1998). As a predictor of yearling count density we used the conspecific basal area per hectare above the sampling threshold (dbh ≥ 10 cm).

We fitted a joined hurdle regression model for *Fagus* and *others*, where the species-specific yearly seedling recruitment rate r is a function of the local and conspecific basal area BA. All other yearly seedling input, i.e. appearing seedlings due to long-distance or temporal dispersal, is included as an intercept k. The hurdle model is implemented as a two-component mixture where the probability density D is the sum of a Bernoulli distribution and a zero-truncated Negative Binomial distribution (ZTNB), parameterized with mean and precision ϕ, such that:

$$\hat{S}_{ps} = k_s + r_s\, BA_{ps} \tag{S1}$$

$$\hat{\Sigma}_p = \sum_{s=1}^{2} \hat{S}_{ps} \tag{S2}$$

$$d_p = \text{Bernoulli}[1 \mid \text{logit}^{-1}(\theta_a + m\hat{\Sigma}_p)] \tag{S3}$$

$$D_{ps} = \begin{cases} d_p & \text{if } S_{ps} = 0 \\ (1 - d_p)\ \text{ZTNB}(S_{ps} \mid \hat{S}_{ps} A_a, \phi_a) & \text{if } S_{ps} > 0 \end{cases} \tag{S4}$$

where S_{ps} are the observed and $\hat{S}_{ps}$ the predicted seedling density, which are a function of intercept k and conspecific basal area BA_{ps} with slope r, and where variables vary with plot p and species s and plots can have one of the sampling area levels a. The Bernoulli density d_p describes the probability of observing no seedlings, which has an inverse logit-transformed linear predictor dependent on the latent total seedling density on a plot across species $\hat{\Sigma}_p$ with slope m, but also on the factor sampling area (parameter θ_a). The hurdle model with a separate process for zero observations was chosen to account for the deflated zeroes with small sampling areas and inflated zeroes with great sampling areas. A deflation of zeroes at smaller sampling areas that could

not be explained by a Negative Binomial distribution was likely a product of the sampling protocol, where the sampling area was increased in steps when the total seedling density was too small, depending on field estimates (see Section Appendix A.1.2). Because the total seedling density $\hat{\Sigma}_p$ is included in the linear predictor for the probability of observing a zero d_p, other than in a classical hurdle model, the zeroes in the data also inform the predicted seedling density $\hat{S}_{ps}$. The zero-truncated Negative Binomial distribution, which describes the non-zero counts, is parameterized with mean $\hat{S}_p$ multiplied by an offset for the sampling area $\hat{A}_a$ and precision ϕ_a, which also varies with the levels of sampling area.

The model was fitted with weakly informative, normally-distributed priors for log-transformations of the parameters of interest $\log r \sim \mathcal{N}(5,10)$, $\log k \sim \mathcal{N}(5,10)$—and regularizing priors $\theta \sim \mathcal{N}(0,2)$, $m \sim \mathcal{N}(0,2)$, and the half-normal $\frac{1}{\sqrt{\phi}} \sim \mathcal{H}(1)$. The resulting posterior distribution of the basal area-dependent seedling recruitment rate $\log r$ was propagated as a prior for the corresponding parameter in the JAB model (Section **??**; Tables S5 and **??**).

Appendix B Code repository

All software to reproduce the study, including the JAB model, has been made openly available at `https://doi.org/10.5281/zenodo.8032461`.

Appendix C Data repository

The data supporting the findings of this study have been made available by the authors at `https://doi.org/10.5061/dryad.3ffbg79pv`.

Appendix D Supplementary Tables

Table S1: Scope of the two NFIs and plot selection.

	German NFI	Slovakian NFI
No. of surveys	3	1
... years	1986–1989, 2000–2003, 2011–2013	2015–2016
No. of plots	61666	1412
... after selection	1000	1402
No. of clusters	21574	.
... after selection	1000	.

Table S2: Size classes for saplings counts and corresponding radii of sampling circles in the three surveys of the German NFI (main years 1987, 2002, 2012). The size classes up to dbh 7 cm, which were consistently sampled across all three surveys, were assigned to the size class J in fitting the JAB model.

	1987	2002	2012
height 20–50 cm	1 m	1 m	1 m
height 50–130 cm	2 m	1.75 m	2 m
height 130–∞ cm and dbh 0–5 cm	2 m	1.75 m	2 m
dbh 5–6 cm	4 m	1.75 m	2 m
dbh 6–7 cm	4 m	1.75 m	2 m
dbh 7–8 cm	4 m	.	.
dbh 8–9 cm	4 m	.	.
dbh 9–10 cm	4 m	.	.

Table S3: Taxa composition of the German NFI on the selected plots. All observed taxa are listed, ranked by the mean basal area per plot. In addition, the mean percentage of the total plot basal area is given.

	Taxon	Mean basal area [m^2 ha^{-1}]	Mean percentage of the basal area
1	Fagus sylvatica	6.174	24.492%
2	Picea abies	5.084	19.730%
3	Pinus sylvestris	4.139	13.145%
4	Quercus petraea	2.692	10.027%
5	Quercus robur	1.293	4.436%
6	Pseudotsuga menziesii	0.779	3.366%
7	Larix decidua	0.767	3.073%
8	Carpinus betulus	0.797	2.825%
9	Acer pseudoplatanus	0.572	2.515%
10	Betula pendula	0.521	2.515%
11	Fraxinus excelsior	0.597	2.309%
12	Abies alba	0.533	2.176%
13	Quercus	0.623	2.158%
14	Prunus avium	0.224	0.949%
15	Tilia	0.208	0.794%
16	Larix	0.246	0.786%
17	Quercus rubra	0.146	0.639%
18	Populus tremula	0.133	0.427%
19	Alnus glutinosa	0.165	0.386%
20	Castanea sativa	0.123	0.364%
21	Acer platanoides	0.061	0.319%
22	Alnus	0.115	0.309%
23	Acer campestre	0.074	0.304%
24	Salix	0.049	0.278%
25	Robinia pseudoacacia	0.107	0.203%
26	Populus	0.074	0.189%
27	Larix kaempferi	0.076	0.188%
28	Pinus nigra	0.068	0.179%
29	Sorbus aucuparia	0.035	0.133%
30	other coniferous (German NFI)	0.035	0.127%
31	Sorbus	0.04	0.110%
32	other Abies (German NFI)	0.024	0.104%
33	Aesculus hippocastanum	0.025	0.067%
34	Ulmus	0.021	0.059%
35	other deciduous (German NFI)	0.015	0.048%
36	Pinus strobus	0.012	0.047%
37	other Acer (German NFI)	0.01	0.041%
38	Ilex aquifolium	0.01	0.031%
39	Populus nigra	0.014	0.028%
40	Abies grandis	0.005	0.027%
41	Prunus padus	0.007	0.026%
42	Sorbus aria	0.005	0.018%
43	other Pinus (German NFI)	0.005	0.017%
44	Thuja	0.004	0.014%
45	Sorbus torminalis	0.003	0.008%
46	other Picea (German NFI)	0.001	0.007%
47	Juglans	0.002	0.004%

Table S4: Taxa composition of the Slovakian NFI on the selected plots. All observed taxa are listed, ranked by the mean basal area per plot. In addition, the mean percentage of the total plot basal area is given.

	Taxon	Mean basal area [m^2 ha^{-1}]	Mean percentage of the basal area
1	Fagus sylvatica	9.102	27.859%
2	Picea abies	7.578	21.290%
3	Quercus petraea	2.54	8.267%
4	Pinus sylvestris	1.92	6.325%
5	Carpinus betulus	1.437	6.316%
6	Abies alba	1.225	3.551%
7	Quercus cerris	0.816	2.768%
8	Betula pendula	0.507	2.600%
9	Acer pseudoplatanus	0.776	2.405%
10	Robinia pseudoacacia	0.519	2.231%
11	Larix decidua	0.638	1.937%
12	Alnus glutinosa	0.508	1.628%
13	Fraxinus excelsior	0.473	1.622%
14	Salix caprea	0.263	1.326%
15	Acer campestre	0.259	1.143%
16	Cerasus avium	0.177	0.882%
17	Populus tremula	0.201	0.864%
18	Salix alba	0.191	0.815%
19	Tilia cordata	0.186	0.675%
20	Alnus incana	0.147	0.591%
21	Quercus robur	0.157	0.556%
22	Pinus nigra	0.153	0.356%
23	Pyrus pyraster	0.048	0.340%
24	Acer platanoides	0.094	0.311%
25	Malus sylvestris	0.043	0.286%
26	Populus x canadensis cv. I 214	0.14	0.265%
27	Prunus domestica	0.024	0.252%
28	Populus nigra	0.189	0.207%
29	Sorbus aucuparia	0.036	0.195%
31	Morus alba	0.021	0.174%
32	Pinus cembra	0.024	0.168%
33	Quercus rubra	0.045	0.165%
34	Sorbus aria	0.046	0.150%
35	Populus x canadensis cv. Robusta	0.011	0.143%
37	Ulmus minor	0.02	0.135%
38	Ulmus glabra	0.034	0.132%
39	Tilia platyphyllos	0.042	0.129%
40	Quercus pubescens	0.028	0.116%
41	Juglans regia	0.007	0.082%
42	Ulmus laevis	0.006	0.071%
44	Castanea sativa	0.028	0.069%
45	Sorbus torminalis	0.015	0.053%
46	Pseudotsuga menziesii	0.016	0.048%
47	Acer negundo	0.011	0.027%
48	Fraxinus ornus	0.005	0.025%
49	Juglans nigra	0.002	0.023%
50	Salix fragilis	0.008	0.014%
51	Celtis occidentalis	0.004	0.013%
52	Fraxinus angustifolia	0.002	0.007%
53	Acer tataricum	0.001	0.005%
54	Betula pubescens	0.001	0.004%
55	Prunus mahaleb	<0.001	0.001%
56	Taxus baccata	<0.001	0.001%

Table S5: Model parameters of prior inference on seedling regeneration r.

		mean	median	sd	q5	q95	convergence (r)	bulk ESS
Fagus	$\log r$	4.2234	4.2262	0.1287	4.0083	4.4302	1.0006	2 855.86
	$\log k$	6.0833	6.0852	0.1904	5.7679	6.3930	0.9999	3 748.85
others	$\log r$	3.0499	3.0621	0.1993	2.6978	3.3468	0.9995	2 951.27
	$\log k$	6.6571	6.6593	0.1276	6.4407	6.8607	1.0004	3 016.92
common	$\operatorname{logit} m$	-4.7850	-4.7356	0.7356	-6.0725	-3.6619	1.0004	3 090.75
	$\operatorname{logit} \theta_1$	-0.4898	-0.4946	0.1387	-0.7179	-0.2618	0.9999	5 257.69
	$\operatorname{logit} \theta_2$	-0.6620	-0.6597	0.2300	-1.0475	-0.2929	1.0010	5 166.38
	$\operatorname{logit} \theta_3$	-0.6581	-0.6590	0.0965	-0.8148	-0.5016	1.0002	4 917.66
	$\operatorname{logit} \theta_4$	-0.6537	-0.6532	0.1421	-0.8951	-0.4200	1.0012	5 323.25
	$\operatorname{logit} \theta_5$	0.6506	0.6511	0.0503	0.5679	0.7319	1.0012	5 427.17
	ϕ_1	0.0175	0.0174	0.0021	0.0142	0.0212	1.0007	4 312.37
	ϕ_2	0.0637	0.0625	0.0130	0.0448	0.0870	0.9999	5 940.02
	ϕ_3	0.0477	0.0473	0.0065	0.0379	0.0592	1.0007	4 244.71
	ϕ_4	0.1637	0.1603	0.0338	0.1129	0.2239	0.9994	4 966.71
	ϕ_5	0.5898	0.5879	0.0623	0.4923	0.6985	1.0006	4 354.77

Table S6: Estimates of the thin plate spline regression for interpolation of the background basal area $\mathfrak{B}$ of *Fagus* and *others*.

Fagus sylvatica

A) parametric coefficients	Estimate	Std. Error	t-value	p-value
(Intercept)	0.1436	0.0308	4.6584	< 0.0001
**B) smooth terms**	**edf**	**Ref.df**	**F-value**	**p-value**
s(Y,X)	120.0256	199.0000	1411.8250	< 0.0001

others

A) parametric coefficients	Estimate	Std. Error	t-value	p-value
(Intercept)	2.3541	0.0200	117.4778	< 0.0001
**B) smooth terms**	**edf**	**Ref.df**	**F-value**	**p-value**
s(Y,X)	65.6964	199.0000	540.0728	< 0.0001

Appendix E Supplementary Figures

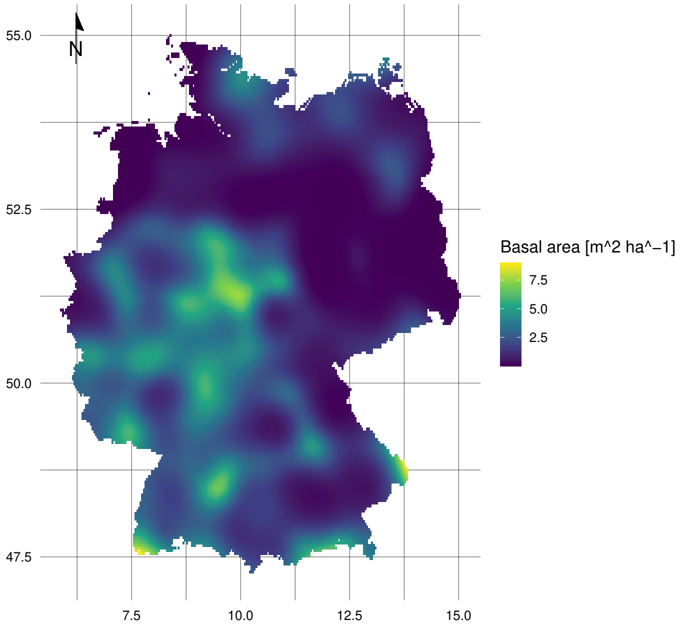

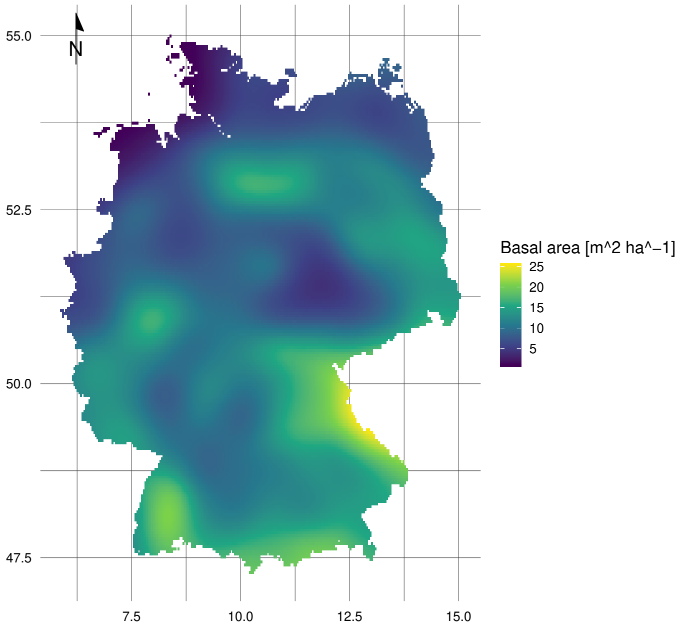

Figure S1: Spatial basal area smoothing splines, which were used as a proxy for background abundance for Fagus (top) and others (bottom) in Germany. For methodological details, see Section Appendix A.3.

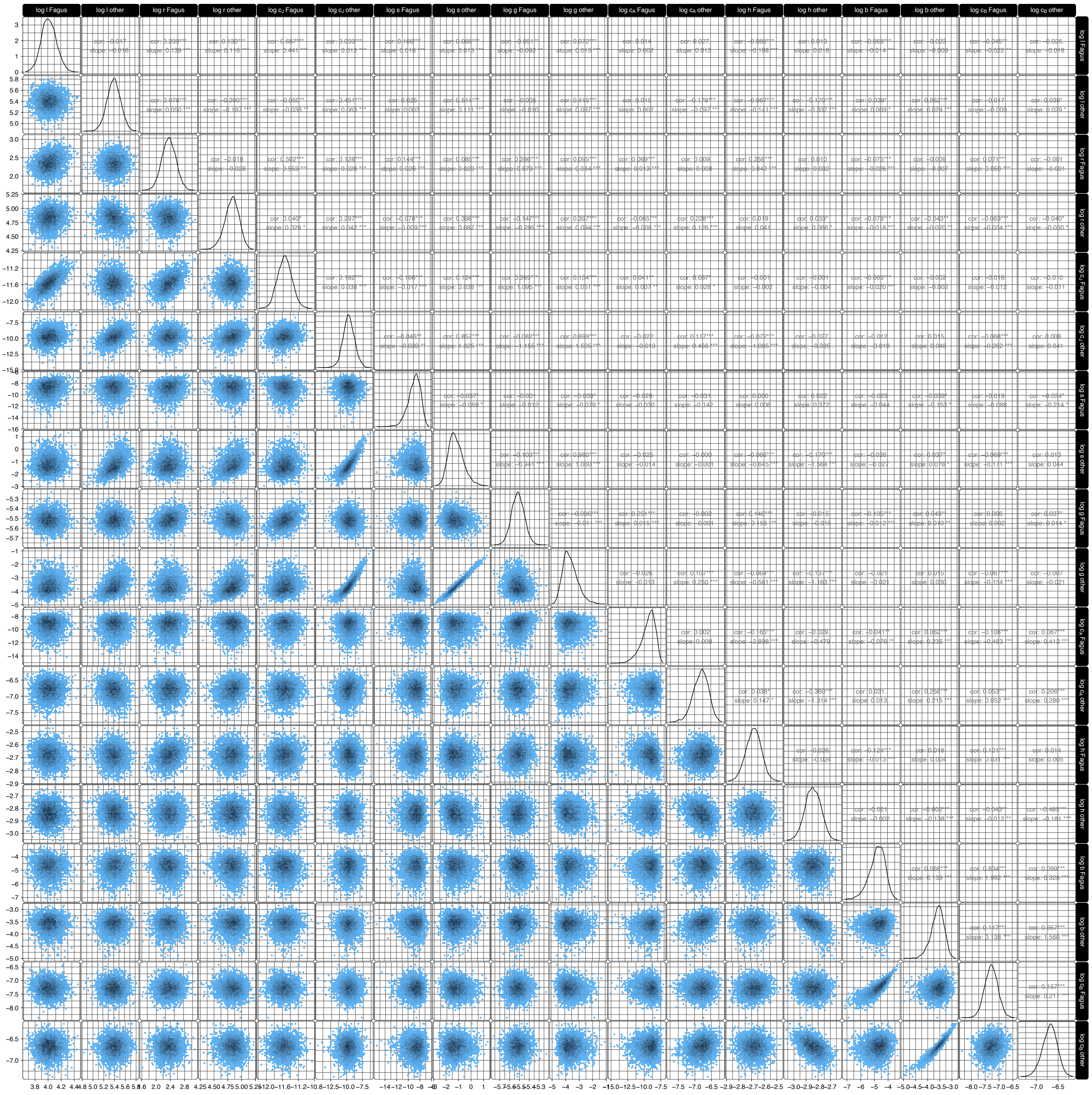

Figure S2: Paired scatter plots of posterior parameter distributions. The lower triangle shows the correlations of the posterior samples, where hexagonal bins are colored by the density of MCMC samples. The diagonal shows the marginal posteriors. The upper triangle quantifies the overall correlation and the slope of a linear regression between the parameters.

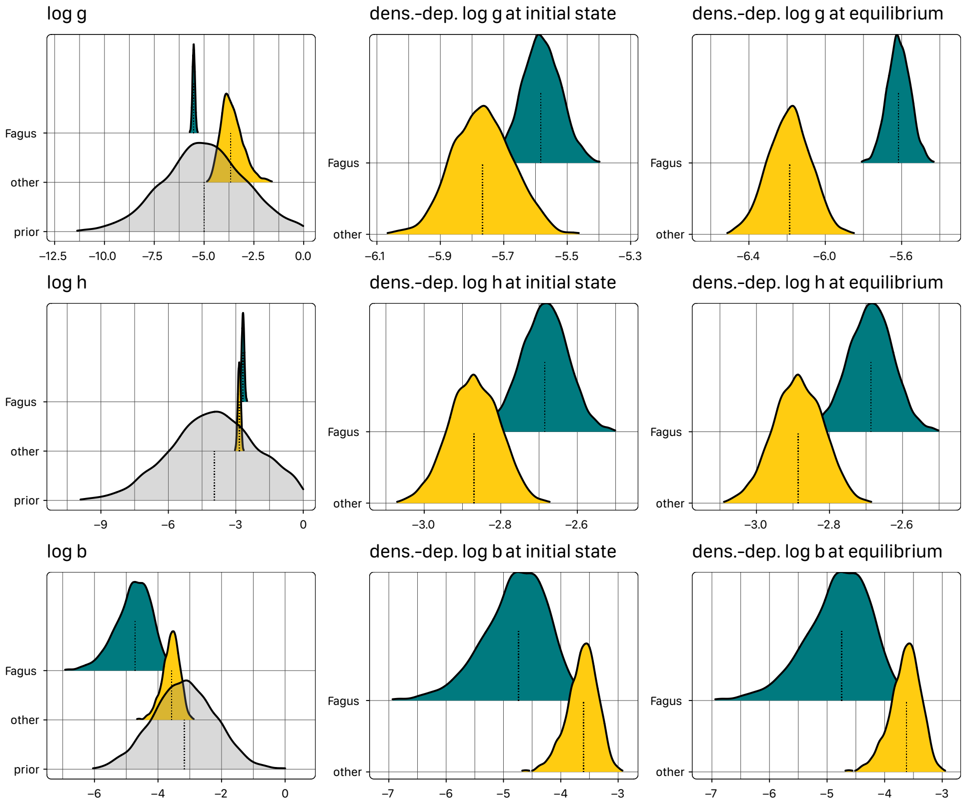

Figure S3: Marginal posterior distributions for parameters g, h, and b with the common priors, as in Figure **??**. In addition, the corresponding density-dependent terms $\frac{g}{s\,\mathrm{sum}(BA)+c_J\,\mathrm{sum}(J)}$, $\frac{h}{c_A\,\mathrm{sum}(BA)}$, and $\frac{b}{c_B\,\mathrm{sum}(BA)}$ are provided. These terms were calculated with the sums of J and BA at both the initial state and competitive equilibrium.

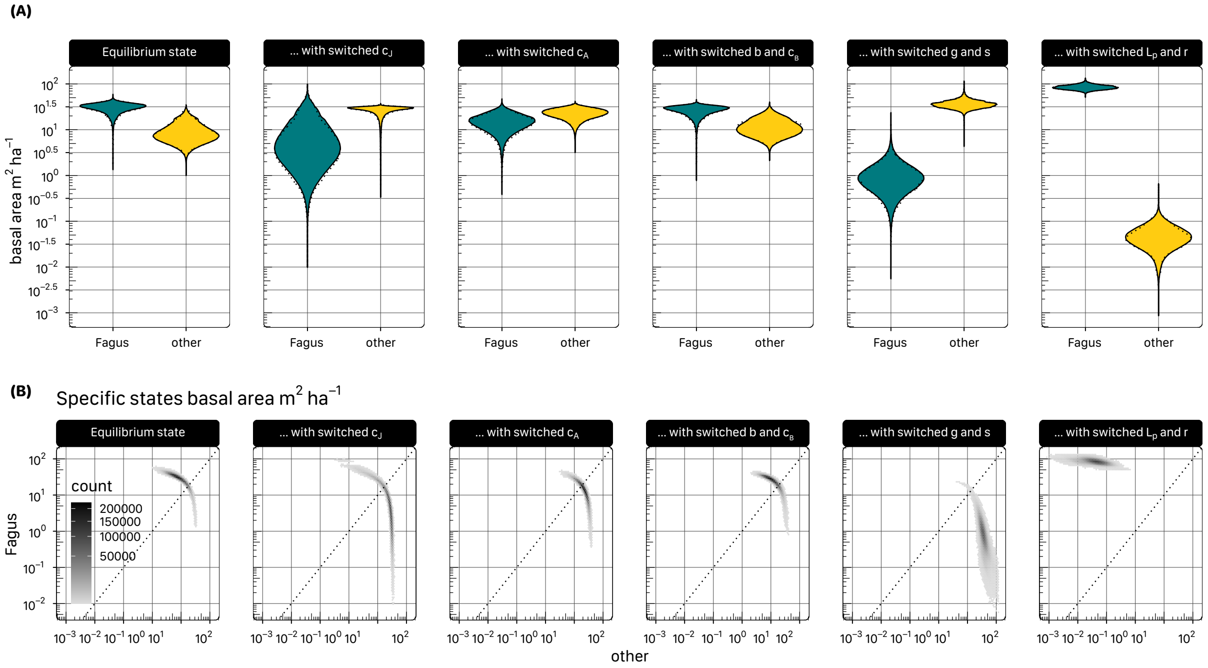

Figure S4: Equilibrium state and counterfactual equilibria as in Figure **??** but with additional parameters switched between species.

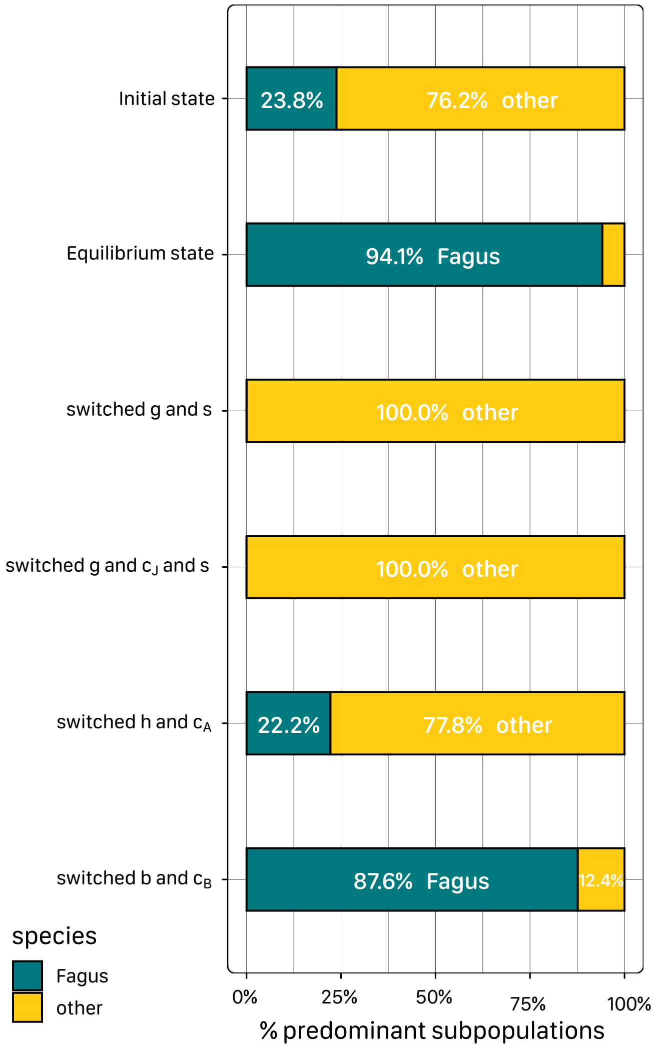

Figure S5: Fraction of posterior cases where either *Fagus* or *others* are the predominant species, as in Figure **??**. Here, in contrast to Figure **??**, the counterfactual equilibria are generated with joint switches of those parameters, that are part of one density-dependent term.

References

Berger, A., Gschwantner, T., and Schadauer, K. (2020). The effects of truncating the angle count sampling method on the Austrian National Forest Inventory. *Annals of Forest Science*, 77(1):16.

Bohn, U. and Gollub, G. (2006). The Use And Application Of The Map Of The Natural Vegetation Of Europe With Particular Reference To Germany. *Biology and Environment: Proceedings of the Royal Irish Academy*, 106B(3):199–213.

Ellenberg, H. (1963). *Vegetation Mitteleuropas Mit Den Alpen. In Kausaler, Dynamischer Und Historischer Sicht.*, volume IV/2 of *Einführung in Die Phytologie.* Ulmer, Stuttgart, first edition.

Geudens, G., Staelens, J., Kint, V., Goris, R., and Lust, N. (2004). Allometric biomass equations for Scots pine (*Pinus sylvestris* L.) seedlings during the first years of establishment in dense natural regeneration. *Annals of Forest Science*, 61(7):653–659.

Kangas, A. and Maltamo, M. (2006). *Forest Inventory. Methodology and Applications.* Number 10 in Managing Forest Ecosystems. Springer, Dordrecht.

Riedel, T., Hennig, P., Kroiher, F., Polley, H., Schmitz, F., and Schwitzgebel, F. (2017). *Die Dritte Bundeswaldinventur (BWI 2012). Inventur- Und Auswertemethoden.* Thünen-Institut, Braunschweig.

Šebeň, V. (2017). *Národná Inventarizácia a Monitoring Lesov SR 2015-2016 (Slovak National Forest Inventory). Informácie, Metódy, Výsledky (Information's, Methods, Results).* National Forest Centre, Zvolen (Slovakia).

Signorell, A. (2021). DescTools: Tools for Descriptive Statistics. https://CRAN.R-project.org/package=DescTools.

Tomppo, E., Gschwantner, T., and for Common, M. L. P. (2010). *National Forest Inventories.* Springer, Heidelberg Dordrecht London New York.

Welander, N. T. and Ottosson, B. (1998). The influence of shading on growth and morphology in seedlings of Quercus robur L. and Fagus sylvatica L. *Forest Ecology and Management*, 107:117–126.

Wood, S. (2021). Mgcv: Mixed GAM Computation Vehicle with Automatic Smoothness Estimation. https://CRAN.R-project.org/package=mgcv.
